# Carvone-Rich Essential Oils and Their Agrobiological Interactions: A Review

**DOI:** 10.3390/molecules31040579

**Published:** 2026-02-07

**Authors:** Agnieszka Krajewska, Grace Azeez, Asgar Ebadollahi, Danuta Kalemba, Agnieszka Synowiec

**Affiliations:** 1 Department of Biotechnology and Food Science, Lodz University of Technology, 90-530 Lodz, Poland; agnieszka.krajewska@p.lodz.pl; 2 Department of Agroecology and Plant Production, University of Agriculture in Kraków, Aleje Mickiewicza 21, 31-120 Kraków, Poland; grace.azeez@student.urk.edu.pl (G.A.); agnieszka.synowiec@urk.edu.pl (A.S.); 3 Moghan College of Agriculture and Natural Resources, University of Mohaghegh Ardabili, Ardabil 56199-36514, Iran; asgar.ebadollahi@gmail.com

**Keywords:** essential oils, carvone, *Mentha spicata*, *Carum carvi*, *Anethum graveolens*, plant pathogens, antifungal activity, insecticidal activity, allelopathy, phytotoxicity

## Abstract

Carvone-rich essential oils (EOs), and carvone specifically, exhibit a broad spectrum of protective effects against major agricultural threats. They display strong antifungal and moderate antibacterial effects, effectively inhibiting numerous phytopathogenic fungi. EOs exhibit significant insecticidal, acaricidal, and repellent activity against various insects and mites, and some EOs are highly effective against agricultural nematodes, suppressing mobility and egg hatching. Crucially, the EOs demonstrate a strong capacity to suppress the germination and initial growth of different weed species, highlighting their viability as natural herbicides. This review analyzes the chemical composition, biological effects, and potential agricultural applications of carvone and carvone-rich essential oils, primarily sourced from *Mentha spicata* (Lamiaceae), *Carum carvi* (Apiaceae), and *Anethum graveolens* (Apiaceae). The biological activity of these EOs is significantly influenced by their specific composition, which varies among plant species and chemotypes. While EOs’ inherent volatility limits direct field application, this challenge is being successfully addressed by innovative formulation technologies, such as nanoemulsification and encapsulation, which enhance stability, bioavailability, and targeted delivery. In conclusion, carvone-rich EOs offer effective, environmentally low-risk agents for the integrated management of pathogens, pests, and weeds in sustainable agriculture. They help reduce reliance on synthetic chemicals and minimize the potential for resistance development.

## 1. Introduction

In the 21st century, the world is undergoing a significant shift from highly industrialized agroecosystems to agroecological systems, which explore the potential of the complexity and interconnections between agro-biocenosis and the agro-environment. In the European Union, this agricultural shift is strongly promoted and implemented through regulations that promote green techniques and ban synthetic pesticides, thereby relegating them to a marginal role [1,2]. While synthetic pesticides remain the primary method for controlling agricultural pests, their prolonged and widespread use has led to numerous adverse effects. These include environmental pollution, risks to human health, harmful effects on non-target organisms such as predators and parasitoids, the development of pesticide resistance, and secondary pest outbreaks [3,4]. Effective alternatives to synthetic pesticides include plant secondary metabolites, e.g., essential oils (EOs). The EOs are multicomponent mixtures of plant volatiles, obtained by steam- or hydrodistillation of different plant parts. EOs produced by aromatic plants can support numerous biological activities and play a vital role in the pharmaceutical, cosmetic, food, and agricultural industries. Essential oils and their components, such as carvone, are gaining popularity as consumers seek natural alternatives to synthetic substances. Furthermore, there is growing interest in using essential oils as alternative biocontrol products against plant pests, including phytopathogenic fungi, bacteria, insects, and weeds [5].

Essential oils have become widely used botanical pesticides in sustainable agriculture, with many studies documenting their toxicity to pests and pathogens. A broad view of the issue was presented in Raveau et al. [6], which reviews EOs as potential alternative biocontrol products against plant pathogens and weeds. Other interesting papers describe the EOs of Apiaceae species as sources of plant-based pesticides [7], as biopesticides [8], or as the subject of structure–activity relationships governing EOs’ phytotoxic effects [9]. One of the promising and intriguing major constituents found in the EOs of various plant species, with significant biological activity and underexplored in recent reviews, is carvone. What is interesting is that carvone is usually present in nature as a specific enantiomer, (*R*)- or (*S*)-carvone. The most important (*R*)-carvone-rich EO is spearmint oil (*Mentha spicata* L., Lamiaceae), while (S)-carvone is the main compound in caraway (*Carum carvi* L., Apiaceae) oil and dill (*Anethum graveolens* L., Apiaceae) oil.

In this paper, a critical review of the chemical composition of carvone-rich EOs and their agrobiological and pest control potential is presented, based on the recent scientific literature.

## 2. Research Methodology

This study collected qualitative and quantitative literature data using a semi-structured method based on the narrative review approach [10]. The literature on carvone and carvone-rich essential oils published between 2000 and 2025 was collected using scientific databases such as SciFinder, Scopus, Wiley Online, SpringerLink, ScienceDirect, and PubMed. The search incorporated keywords including “essential oils”, “carvone”, “*Mentha spicata*”, “*Carum carvi*”, “*Anethum graveolens*”, “plant pathogens”, “antifungal activity”, “insecticidal activity”, “acaricidal activity”, “nematicidal effect”, “allelopathy”, and “phytotoxicity”. This review examines the application of essential oils (EOs) for pest control in plants across both growth and post-harvest stages, as reported in the scientific literature. The review also briefly addresses new formulations designed to enhance the stability and efficacy of EOs. Studies focusing on foodborne pathogenic bacteria are excluded. Only articles reporting the composition of carvone-rich EOs (containing more than 30% of carvone) were included. Research employing methods that do not permit result comparison, particularly those using the disk diffusion test, was omitted. Older articles were cited only in exceptional cases.

## 3. Characteristics of Carvone and Carvone-Rich Essential Oils and Their Plant Representatives

**Figure 1:**
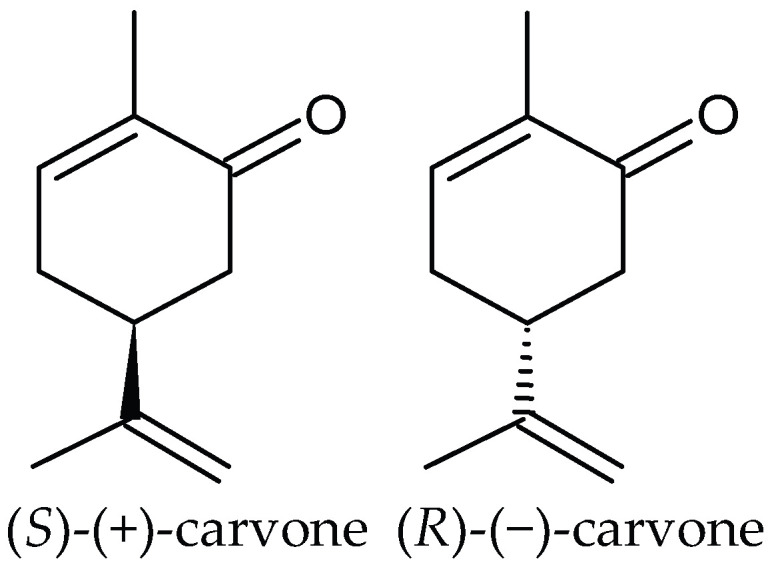
Structure of carvone (*S*)-(+)- and (*R*)-(−)-enantiomers [11].

Carvone (*p*-mentha-6,8-dien-2-one, C_10_H_14_O) is an optically active monoterpene ketone (Figure 1) widely distributed in the plant world with broad biological activity. It is a constituent found in many essential oils as a dominant compound (e.g., *C. carvi*, *A. graveolens*, *M. spicata*, *Lippia alba*) as well as a trace ingredient in other oils. Due to its single chiral center, carvone exists in two enantiomeric forms: (*S*)-(+)-carvone and (*R*)-(−)-carvone. Carvone enantiomers have the same physical and chemical properties, except for the sign of their optical rotation. However, the enantiomers may differ in terms of biological activity. In particular, they have a significantly different scent. (*S*)-carvone has a spicy aroma with rye notes like caraway seeds, whereas (*R*)-carvone has a sweetish, minty smell like spearmint leaves. The odor of both isomers is of medium strength. Carvone has a boiling point of approximately 231 °C, a density of around 0.959 g/mL at 25 °C, and is practically insoluble in water but soluble in alcohol and nonpolar environments [11].

The three most important carvone-rich EOs are obtained from raw materials that are well-known culinary spices worldwide: the fruits of two Apiaceae family members, *Carum carvi* L. and *Anethum graveolens* L., and the herb *Mentha spicata* L. (family Lamiaceae). Commercial caraway oil (CCEO) and dill oil (AGEO) contain similar levels of the two main optically active constituents, namely (*S*)-carvone and (*R*)-limonene. *A. graveolens* also provides EOs from its leaves and flowers that contain significantly lower amounts of carvone. Spearmint oil (MSEO) contains (*R*)-carvone.

**Figure 2:**
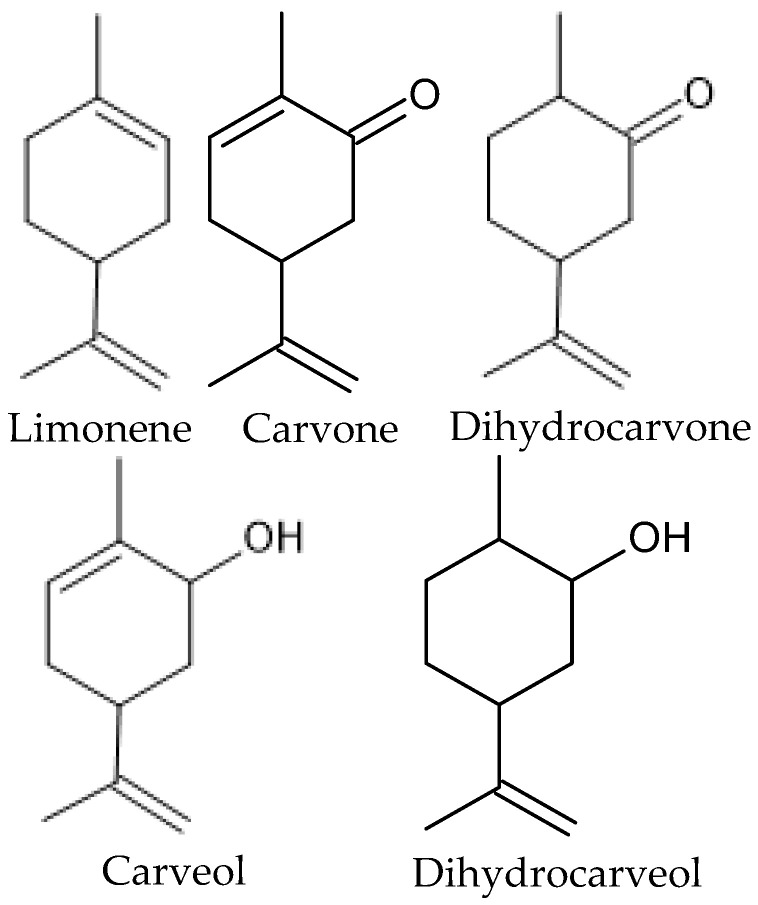
The main constituents of *Carum carvi* L. essential oil [11].

Caraway (*Carum carvi* L.) is a biennial medicinal plant and a common spice in Europe, Asia, and Africa. Of the approximately 25 *Carum* species, only *C. carvi* is economically significant and is grown and used worldwide mainly for its fruit. Caraway fruit is a pharmacopeial material with many pharmacological properties [12]. The crescent-shaped light to dark brown fruits have a single seed with a warm, sweet, and slightly sharp flavor [13]. The most important active ingredient of caraway fruit is the essential oil, known as caraway oil (CCEO), obtained by the hydrodistillation of crushed fruit, with a yield of 3–7%. According to the European Pharmacopoeia 10th edition, the main components of caraway oil are: carvone (50–65%), limonene (30–45%), trans-carveol (max. 2.5%), trans-dihydrocarvone (max. 2.5%), and myrcene (0.1–1%) (Figure 2). (*S*)-carvone and (*R*)-limonene are present in high enantiomeric purity [14].

Seventeen selected caraway genotypes, originating from European botanical gardens, as well as one cultivar and two authors’ strains, have shown little variation in EO yield (3.4–5.2%) and in the content of the two main compounds (carvone 53–68%, limonene 28.5–40.5%) [15]. A similar composition was reported for caraway cultivated in Tunisia (carvone 76.4%, limonene 19.5%), Germany (77.4%, 16.2%), and Egypt (61.6%, 29.1%, respectively) [16]. Reversed proportions of two main CCEO compounds were observed in three ecotypes of annual caraway in Serbia: carvone (27.4–44.5%), limonene (54–70.3%) [17]. The content of EO in biennial caraway (3.9–5%) is higher than in annual caraway (2.8–3.3%) [18]. The yield of CCEO depends strongly on the form of fruit. Proper preparation of the seed and fruit for hydrodistillation includes crushing the plant material. This step is often omitted, resulting in yields of 1.4% [19,20] or even 0.48% [16]. Frequent minor constituents of the caraway oil are cis- and trans-dihydrocarvone, cis- and trans-dihydrocarveol, perillaldehyde, myrcene, γ-terpinene, and other mono- and sesquiterpene hydrocarbons. Minor compounds in total amounted to 3–4% in annual and 1.5–2.5% in biennial plants [18].

Caraway oil is sometimes confused with cumin oil isolated from *Cuminum cyminum* L. Both plants belong to the same Apiaceae family, and their fruits are used as culinary spices and in traditional therapies. Both fruits are rich sources of EOs, but their compositions differ significantly. In some papers, instead of declared caraway oil, cumin oil was researched [21,22,23,24] because the EOs tested contained mainly cuminaldehyde and γ-terpinene, which are the major components of EO from the fruits of *C. cyminum* [25,26]. Such papers are omitted in this review. To the contrary, other authors named EO from *C. carvi* as cumin oil [27]. What is more, these mistakes are repeated in reviews and other papers while discussing the antifungal activity. The structures of the main AGEO components are presented in Figure 2.

Dill (*Anethum graveolens* L.), a biennial or annual herb of the Apiaceae family, is native to Southwest Asia and Southeast Europe and has been cultivated since ancient times for its economic and medicinal value [18,28]. The only species in the genus *Anethum* is *A. graveolens* L. Its variant, known as East Indian dill or Sowa (*A. graveolens* var. sowa Roxb. ex, Flem., syn. *A. sowa*), is found in India and is grown as a cold-weather crop in the Malay Archipelago, Japan, and the Indian subcontinent for its leaves. The yellow blossom turns into umbels [29]. Dill has pseudo-seeds, the halves of schizocarps, which are tiny, dry fruits. The fruits have a flavor that is a little like caraway. Compared to caraway, the fruits are lighter, flatter, and smaller. They also have a nice and delicious scent [30]. Dill oil is obtained by hydrodistillation of the seed/fruit, flower, and leaf [28,31]. Seed and fruit represent the same raw material, as with caraway, where the small dry fruits are often mistaken for the seeds. The most popular commercial dill oil is obtained from the fruit. EO distilled from dill foliage differs markedly in yield and composition from dill seed oil. In this paper, the dill fruit EO (AGEO) is considered.

Many authors have reported the chemotypes of dill based on volatile compositions across plant parts and developmental stages. The oldest available and still relevant work on this subject analyzed the composition of pentane extracts from the seeds of 35 dill cultivars [32]. The presence or absence of carvone, myristicin, and dillapiole distinguished three chemotypes. The major components of the most numerous chemotypes (27 cultivars) were carvone (43.7–57.7%) and limonene (39.5–50.7%). Limonene (36.9–46.7%), carvone (17.8–45.6%), myristicin (0.2–20.3%), and dillapiole (8.0–22.3%) were the main components of five samples whereas carvone (25.1–47.4%), limonene (31.0–40.9%), and dillapiole (6.3–31.8%) were the main components of three samples [32]. In addition to these three chemotypes, 66 fruit seeds yielded some transition chemotypes [33].

**Figure 3:**
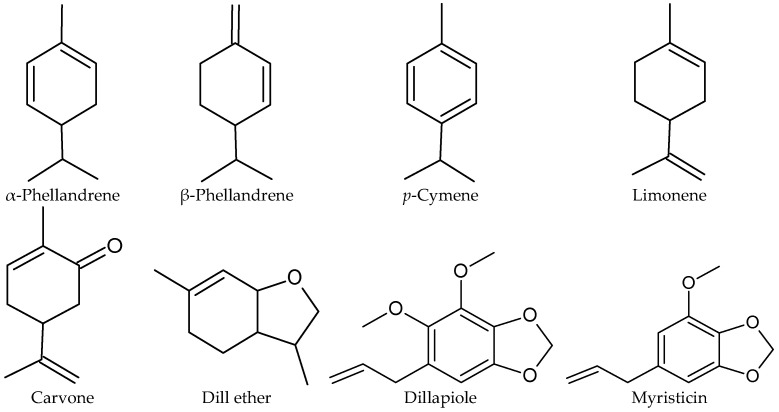
The main constituents of *Anethum graveolens* L. essential oil [11].

Fruits of 26 commercially available dill cultivars yielded 3.4–4% AGEO. The content of components was presented in relation to the mass of fruit and, for the two main compounds, was 10.7–13.0 mg/g of carvone and 12.0–14.2 mg/g or 14.9–17.8 mg/g of limonene, depending on the harvest year. Apiole and myristicin were absent in most samples, but were detected at 0.2–11% in dill chemotypes where they were present. Minor components such as α-phellandrene, dill ether, and cis- and trans-dihydrocarvone were below 10% with an average of 7% [18]. The structures of the main AGEO components are presented in Figure 3.

Seven European dill cultivars belonged to carvone (81.4–90.0%)/limonene (9.6–18.0%) chemotypes, and the local Egyptian cultivar contains carvone (56.6%), limonene (18.8%), dillapiole (15.7%), and piperitone (7.4%) [34]. The same authors reported carvone (56.6%, 62.5%), limonene (18.8%, 14.6%), and dillapiole (15.7%, 19.5%) as the main compounds in fruit EO, which was isolated with a yield of 3.2% [35,36]. Dill herb at the vegetative stage yielded 0.08% of EO and contained α-phellandrene 46.3%, p-cymene 17.9%, limonene 13.7%, and ß-phellandrene 11.0%. In comparison, herb EO at the flowering stage (yield 1.1%) contained p-cymene (33.4%), dill ether (19.6%), and carvone (13.1%) [35].

Chahal et al. [31] in their review presented more examples of AGEO chemotypes, e.g., several containing 5–16% of dihydrocarvone isomers. However, it is worth noting that almost all dill fruit EOs contained carvone and limonene as the two main components.

Similar to caraway oil, AGEO is sometimes isolated from the whole fruit rather than crushed fruit, resulting in a very low yield of 0.8% [37].

Spearmint (*Mentha spicata* L.) belongs to the genus *Mentha* (family Lamiaceae) [38,39]. Three of the globally most extensively cultivated and economically important mint species produce essential oils that differ in the main constituents: in peppermint oil (*M. x piperita* L.) and cornmint oil (*M. arvensis* L.) (1*R*, 3*S*, 4*S*)-(−)-menthol is the main compound while in spearmint oil (MSEO) usually (*R*)-carvone dominates. Chemotypes of other naturally occurring mints can also produce carvone-rich EOs, e.g., *M. cardiaca* L., *M. suaveolens* Ehrh., and *M. longifolia* (L.). Huds. [38,40], andare included in this review. On the other hand, *M. spicata* exhibits different chemotypes, including pulegone, piperitone, and piperitenone oxide, which are not described in this review [39,41]. It is important that, among the 77 *M. spicata* EOs listed by Mahendran et al. [42], only the third was the carvone chemotype. This shows how important it is to determine the EO composition (especially in the genus *Mentha*) in all biological studies and confirms the validity of omitting from this review those articles that do not provide the EO composition.

**Figure 4:**
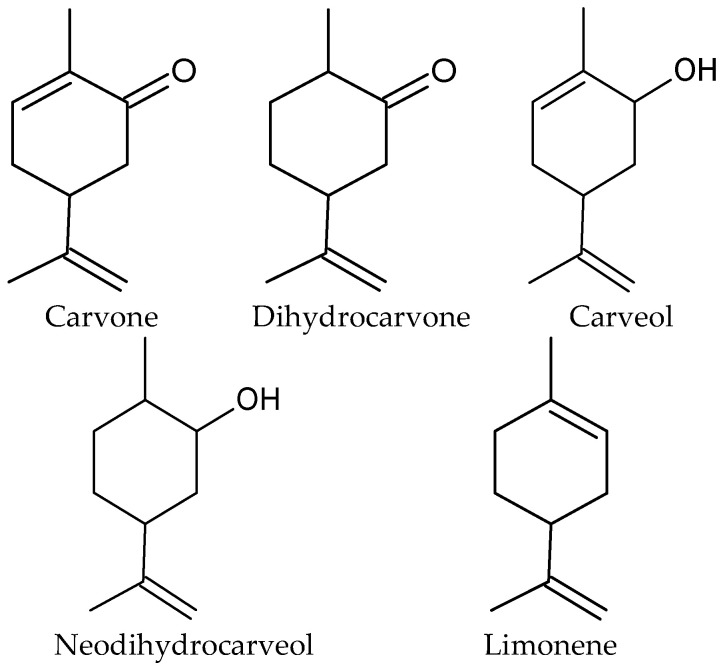
The main constituents of *Mentha spicata* L. essential oil [11].

*M. spicata* is a perennial mainly grown in the USA, preferring wet habitats like riverbanks. It has an erect, often red-flecked stem (50–90 cm), pointed green leaves, and flowers in pseudo-spikes at shoot tops. *M. spicata* has several popular cultivars originating from Europe and North Africa [43]. Spearmint is an aromatic and medicinal plant, and its herb and leaves are widely used in various applications and have been the subject of review papers [44]. Spearmint oil (MSEO) is produced by the hydrodistillation of fresh or dried herbs [42,45] with a yield of 0.23–3.24% [46]. Commercially exploited *M. spicata* plants always contain EO rich in (*R*)-carvone (30–85%) and (*R*)-limonene (5–28%) that are accompanied by smaller amounts of related compounds such as cis- and trans-dihydrocarvone, alcohols dihydrocarveol, cis- and trans-carveol and their acetates, linalool, terpinen-4-ol, 1,8-cineole, menthone, as well as mono- and sesquiterpene hydrocarbons [38,42]. The structures of the main AGEO components are presented in Figure 4.

## 4. Biological Activities

### 4.1. Antifungal and Antibacterial Activity

Research over the past two decades has shown that essential oils (EOs) and their components, such as carvone, exhibit antifungal and antibacterial effects. Studies often use agar diffusion (measuring inhibition zones, IZs) or serial dilution (measuring the minimal inhibitory concentration, MIC; minimal bactericidal/fungicidal concentrations, MBC/MFC; or the inhibition rate, IR). Negative and positive controls are ideal but not always included. However, comparing results across labs is difficult due to differences in test conditions and reporting methods. MIC, MFC, and IR values are more comparable between studies than inhibition zones. [47,48]. For this reason, only selected, well-documented studies that used this latter method will be presented. Often, along with the assessment of antifungal activity, the impact of EO on aflatoxin production is determined.

A broad range of phytopathogenic fungi (Table 1) and only a few phytopathogenic bacteria (Table 2) were tested for their susceptibility to carvone and carvone-rich EOs. Three aspects of antimicrobial activity in in vitro tests are reported in the literature: comparisons of carvone activity with other monoterpenes, comparisons between different EOs and compounds, and new formulations containing active substances. In the case of fungi, anti-aflatoxin activity was also assessed. Sometimes, in vitro tests are accompanied by research in in situ conditions, which are usually referred to as in vivo.

The current status of in vitro research indicates that carvone enantiomers generally show slight differences in antimicrobial activity, with the (*R*)-carvone isomer being more effective than the (*S*)-isomer. However, both carvone isomers are frequently more effective than other monoterpenes such as 1,8-cineole [49], isopulegol [50], or terpinene-4-ol, but less active than thymol [51,52]. For example, carvone has shown total mycelial growth inhibition at 0.8–1% for *Colletotrichum gloeosporioides* and *C. musae*, while successfully inhibiting the growth of *Aspergillus flavus* [50] and *A. niger* [49,53], and also aflatoxin B1 production at very low concentrations [54,55].

Caraway oil (CCEO) often proved more active against fungi than other EOs, e.g., coriander (*Coriandrum sativum*) EO [56], two citronella EOs [57], juniper EO [58], or many EOs [59]. CCEO effectively inhibited pathogens, including various *Aspergillus* species [27,57,60,61,62], *Penicillium* species [63,64,65], *Sclerotium rolfsii* [66], and *Botrytis cinerea* [67]. CCEO, when tested in mixtures with other EOs (clove oil and cumin oil), was more active against *A. niger* (MIC 1–2 mg/mL) than the single oils (MIC 1–3 mg/mL), suggesting a synergistic effect [68].

CCEO and its major constituent, carvone, also exhibited high antibacterial activity against Gram-positive genera, such as *Clavibacter*, and Gram-negative genera, such as *Erwinia* and *Xanthomonas* [25]. The bacteriostatic activity of CCEO and carvone was the same, while carvone showed better bactericidal efficacy and also demonstrated synergistic effects when combined with β-lactams [69].

Two primary chemotypes of dill essential oil (AGEO), characterized by carvone/limonene/dill apiol and carvone/limonene, exhibit nearly identical levels of antifungal activity. AGEO has demonstrated the ability to completely inhibit *A. flavus* growth at concentrations of 1.2–1.25 µL/mL [37,70]. Different methodologies yield varying results; for instance, AGEO achieved 100% inhibition of *A. niger* and *Penicillium citrinum* using the poison food technique, yet showed less than 15% inhibition against *A. niger* when using the inverted Petri plate method [65]. AGEO demonstrated high toxicity against a broad spectrum of common food-biodeteriorating fungi, with many strains suffering 100% inhibition at MIC doses [37]. It also showed promising activity against plant pathogens, including *Alternaria triticina* and *Bipolaris sorokina* [31]. Research on mushroom pathogens indicates that AGEO has the highest median ability among 11 EOs tested against *Lecanicillium fungicola*, while affecting the growth of the mushroom *Agaricus bisporus* at significantly higher concentrations [59]. AGEO inhibited the mycelial growth, sporulation, and germination of seven fungal species [71]. It should be stressed that, in studies testing many EOs, AGEO was among the more effective against many fungal strains [59,72,73].

The antifungal efficacy of AGEO varies significantly depending on the plant part used, with oils derived from fruits and seeds generally demonstrating superior activity compared to those from leaves or herbs [74].

Essential oil isolated from the herb or leaves of *Mentha spicata*, known as spearmint oil (MSEO), has been extensively researched for its chemical composition and biological activity, with recent reviews highlighting its various chemotypes, particularly those rich in carvone [46,75]. MSEO effectively inhibited the growth and aflatoxin B1 production in toxigenic *A. flavus* at concentrations of 1.0 µL/mL and 0.9 µL/mL, respectively. At a MIC value of 1.0 µL/mL, the oil showed broad fungistatic effects against 19 food-deteriorating molds, achieving 100% inhibition in nearly all species except *Aspergillus luchuensis* and *A. terreus* [64]. Similarly, *Mentha cardiaca* EO (carvone 59.6%) was effective against 20 fungal species identified in dry fruits. While most fungi were inhibited at an MIC of 1.25 µL/mL, *Rhizopus stolonifer* proved resistant [76]. Similar resistance in *Rhizopus* spp. was reported by Hussain et al. [77], who noted MIC values up to 157.8 µg/mL. Against *Geotrichum citri-aurantii*, MSEO showed medium activity (31% inhibition at 1000 µL/mL), significantly outperformed by its pure constituent (*R*)-carvone, which achieved 100% inhibition [78]. MSEO demonstrated activity against various fungal pathogens of *Agaricus bisporus*, though it was less effective than oregano or thyme oils [79]. In studies involving MSEO with high carvone (51.7%) and cis-carveol (24.3%) contents, pure carvone and cis-carveol were more effective than the whole oil against *A. niger* and *Botryodiplodia theobromae* [80]. The efficacy of MSEO is highly dependent on laboratory conditions. MIC values for the same EO were 1.0–2.5 μL/mL with ethanol as the solubilizer, but dropped to 0.5–1.5 μL/mL with Tween. At the same time, MICs assessed by the macrodilution method ranged from 3.5 to 5 µL/mL and by the microdilution method from 0.5 to 2.5 µL/mL. By the latter method, the MFC value was 1.5–2.5 µL/mL [81]. In vapor tests, MSEO achieved 100% inhibition of *Verticillium dahliae* at 16 µL/dish [82] and over 90% inhibition of *Rhizopus stolonifer* [83]. (*R*)-carvone generally demonstrates superior antifungal activity compared to (*S*)-carvone or the whole AGEO [78,81]. As a critique, some researchers [77] incorrectly claimed that EOs were more potent than standard drugs; in reality, the EOs required doses roughly 500 times higher to achieve comparable inhibition zones [80].

A few other plant species also produce carvone-rich EOs. The best known are two species of the genus *Lippia*: *L. alba* and *L. scaberrima* (family Verbenaceae). Leaf EOs of the *L. alba* carvone chemotype inhibited fungal growth, with (*R*)-carvone being more effective than EO [84,85]. The EO of aerial parts of *L. scaberrima*, along with carvone enantiomers and limonene, was tested using two methods for its activity against *Botryosphaeria parva* and *C. gloeosporioides* [86], as well as against *Alternaria* sp., *C. gloeosporioides*, and *Lasiodiplodia theobromae* [87]. Fungal strains were isolated from fruits, and the efficacy of EO in preventing fungal diseases was also tested. In a serial dilution on a solid medium, all substances showed very good activity. Two enantiomers of carvone exhibited stronger fungistatic activity than EO, whereas limonene and 1,8-cineole showed little antifungal activity. Additionally, the fungicidal activity of carvone and EO, but not of limonene, was observed [86,87].

Leaf EOs of the carvone (85.9%) chemotype of *Aloysia polystachya* tested by the fumigant method were more effective in inhibiting conidia germination (47.2 µL/L air) than mycelial growth (MIC 71 µL/L air) of *B. cinerea* [88].

Especially interesting were the studies that compared the activity of different carvone-rich EOs. MSEO, as well as its pure compound (*R*)-carvone, completely inhibited the mycelial growth of *P. digitatum* at a concentration of 1000 µL/L, while the EO of *L. scaberrima* at 3000 µL/L. The authors did not report the percentage compositions of the EOs [89]. In the next article of the Regnier team, CCEO, AGEO, MSEO, and *L. scaberrima* EO were included in a study of 18 EOs and seven compounds against 10 strains of six fungal species. Thyme oil and thymol were the most active (100% inhibition at concentration 500–1000 µL/L except for *P. digitatum*), followed by eugenol, carvone isomers, and carvone-rich EOs. MSEO was the most efficient among the four carvone-rich EOs, especially toward *P. digitatum* (1000 µL/L). EOs containing (*R*)-carvone were more effective than those containing the (*S*)-enantiomer [90]. On the other side, among the 15 tested EOs, only vapors of three EOs (thyme, cinnamon bark, and oregano) at the concentration 212 µL/L caused 100% inhibition of the mycelial growth of *Galactomyces citri-aurantii* and *P. digitatum*, while MSEO and CCEO in the tested concentration range (27–212 µL/L) exhibited a slight stimulatory effect [91].

Recently, in vitro research has increasingly been complemented by experiments conducted in real-world settings, referred to as in vivo or in situ methods. In addition, several articles present only in situ studies and include the standard fungicides. On the other side, new formulations with EOs are tested in both types of studies. In situ studies on carvone and carvone-rich EOs, as well as studies with new EO formulations, are shown in Table 1, where the details are reported.

In laboratory conditions, CCEO, in solution or emulsion form, appeared to be effective in inhibiting the growth of *A. flavus* contaminating peanuts and corn flour [92]. The combination of pre-harvest foliar spraying with salicylic acid, followed by post-harvest CCEO coating emulsion, considerably decreased post-harvest chilling injury in stored sweet pepper fruit [67]. CCEO and MSEO were shown to be alternative preservation methods for Jerusalem artichoke tubers during storage, resulting in a lower severity of *Sclerotium* tuber rot, sprouting percentage, and weight loss [61,66]. AGEO was strong at inhibiting green mold on citrus fruit inoculated with *P. digitatum* [73]. Vapors of AGEO were efficient in the reduction in *A. alternata* and *A. niger* on inoculated cherry tomatoes [93] and reduced Rhizopus rot on strawberry and peach fruits, both uninoculated and inoculated [83]. Vapors of the carvone-type EO of *Aloysia polystachya* were efficient in reducing the symptoms of gray mold on cherry tomatoes infected with *B. cinerea* [88]. Vapors of MSEO (1.0 µL/mL air) caused effective protection of chickpea against *A. flavus* [94]. *Mentha viridis* EO (carvone 58%) was shown to be highly effective in reducing the growth of *Fusarium graminearum* and *F. culmorum*, and in the biosynthesis of mycotoxins in inoculated maize seeds [95].

In several studies, the protective effect of coatings enriched with EOs has been demonstrated. Pullulan films containing 8% and 10% CCEO tested as edible coatings on baby carrots during storage significantly reduced the population of *A. niger* inoculated on carrots [61]. MSEO and *L. scaberrima* EOs incorporated at 2500 µL/L into commercial wax coatings provided excellent disease control on oranges treated with *P. digitatum*, comparable to the control treatment with synthetic fungicides [89]. While tested on avocado fruit inoculated with three fungi, the coatings with MSEO and *L. scaberrima* performed equally well, or better, than conventional disease control using synthetic fungicides [87]. Similarly, commercial wax coatings enriched with the EO of *L. scaberrima* were significantly more effective at treating infection by two pathogenic fungi on inoculated mango fruit than coatings alone [86].

A few in situ studies were carried out using carvone-rich EOs–chitosan nanoemulsions as fumigants. CCEO–chitosan nanoemulsion caused efficient protection in inoculated *Withania somnifera* root against fungal infestation by four *Aspergillus* species and *Penicillium purpurogenum* [19]. AGEO–chitosan nanoemulsion preserved rice seeds uninoculated or inoculated by *A. flavus* [37]. Conversely, encapsulation in copper nanoparticles did not enhance AGEO’s activity against *Colletotrichum nymphaeae*; instead, the standard EO remained more effective at inhibiting both mycelial growth and germination [96].

MSEO and carvone encapsulated in two polymers, when added to a box containing kumquats, significantly reduced disease incidence caused by citrus post-harvest pathogens, *P. digitatum* and *Geotrichum citri-aurantii*, for 2 weeks, whereas neat carvone and MSEO were active for only 1 week [97].

Very rarely have carvone-rich EOs been tested under greenhouse and under field conditions. Emulsified CCEO used as a coating of faba bean seeds significantly reduced bean root rot incidence and protected seeds against pathogenic fungi better than the fungicide Rhizolex-T [60]. In another study, the strong antifungal activity and modest antibacterial activity of carvone and the carvone–PGLA composite were demonstrated in a greenhouse test on lettuce and wheat seedlings infected with a carvone–PLGA composite which was more effective [98].

A special example of in situ studies has been conducted using fresh leaves of essential oil-bearing plants. In an in vitro study, volatiles released from the leaves of the carvone chemotype of *M. spicata* were the most effective among seven herbs in inhibiting the growth of *F. oxysporum* and *Pythium aphanidematum* [99]. In the study, under pot culture and field conditions, a significant reduction in the severity of damping-off in tomato crops was observed following exposure to the volatiles of *M. spicata*, with relatively abundant control of *P. aphanidematum* [100].

Many in situ studies showed that treating with EOs reduced aflatoxin levels [19,37,95] or other mycotoxin levels [95].

It is very important that it was proven that the use of EO formulations for the protection of fruit and vegetables does not affect organoleptic parameters [89]; on the contrary, it resulted in improved quality, reduced weight loss, and the retention of firmness and biochemical features, e.g., capsaicin content in sweet pepper fruit [67], and improved visual acceptability of baby carrots [61].

The specific mechanisms by which EOs act on fungal cells remain poorly elucidated. The antifungal targets of EOs and their constituents are mainly the cell wall, plasma membrane, mitochondria, and antioxidant enzymes [101]. In-depth insights into these mechanisms, conducted using carvone and carvone-rich EOs, supported this statement. Das et al. [54] and Wei et al. [55] assessed the antifungal and anti-aflatoxin B1 mechanisms of carvone (isomer not reported) on *A. flavus*, and showed that carvone disrupted the integrity of the cell wall and membrane, induced reactive oxygen species accumulation, caused DNA damage, triggered cell autophagy, and reduced ATP levels, which ultimately led to cell death. Carvone inhibited spore formation of *A. flavus* by suppressing the transcription of spore development-related genes. Similar effects were observed when *A. flavus* was treated by AGEO, CCEO, and *M. cardiaca* EO [76]. The antifungal activity of AGEO results from its ability to disrupt the plasma membrane permeability barrier and reduce mitochondrial enzyme activity, leading to ROS accumulation [70]. CCEO altered the permeability of *A. flavus* cells, leading to increased leakage of essential cellular ions [62]. MSEO disrupted the membrane integrity of *R. stolonifer* [83].

In summary, comparative studies indicate that carvone and carvone-rich EOs exhibit better antifungal activity than many other EOs and their components. However, carvone is less active than thymol, and, consequently, EOs rich in phenols are usually more active than carvone-rich EOs. Usually, both carvone isomers were more effective compared to EOs containing them. It is interesting to note that, in studies with both carvone enantiomers, the (*R*)-isomer showed better antifungal activity than the (*S*)-isomer. Carvone-rich EOs inhibited spore germination and mycotoxin production more effectively than mycelial growth and were more potent toward fungi than bacteria.

**Table 1:** In vitro antifungal activity of carvone and carvone-rich essential oils against phytopathogens.

Fungi	Compound EO: Main Compounds [%] [No. of EOs/Compounds Tested]	Results	Methods	Ref.
*Aspergillus flavus*	carvone	MIC 0.8 μL/mL	Mycelial growth in serial broth dilution, spore germination on solid medium, in situ on corn flour and peanuts	[55]
*Colletotrichum gloeosporioides* *Colletotrichum musae* *Fusarium subglutinans*	(*R*)-carvone [5 compounds]	MIC 1.0% 0.8% >1.0%	Mycelial growth and conidia germination on solid medium Negative control benomyl	[50]
*Alternaria alternata*, *Aspergillus tubingensis*, *Aspergillus carbonarius*, *Fusarium subglutinans*, *Fusarium cerealis*, *Fusarium culmorum*, *Fusarium verticillioides*, *Fusarium proliferatum*, *Fusarium oxysporum*, *Fusarium sporotrichioides*, *Penicillium* sp.	carvone [5 compounds]	EC_50_ 0.028%, EC_90_ 0.08% Mean values for all compounds and fungi	Mycelial growth on solid medium	[52]
*Aspergillus niger* *Fusarium oxysporum* *Penecillium digitatum* *Rhizoctonia solani*	(*R*)-carvone [12 monoterpenes]	EC_50_ 120.0 mg/L 432.5 mg/L 418.0 mg/L 274.0 mg/L	Serial dilution on solid medium Positive control carbendazim	[53]
*Aspergillus flavus*	(+/−)-carvone [25 monoterpenes]	EC_50_ 550 mg/L	Serial dilution on solid medium Positive control carbendazim	[51]
*Aspergillus flavus* *Aspergillus fumigatus* *Fusarium graminearum* *Sclerotinia sclerotiorum*	(−)-carvone and (−)-carvone-PLGA-composite	IR 35% and 60% at 0.25 mg/mL 23 and 100% 28 and 100% 100 and 100%	Mycelial growth inhibition on solid medium at 4 concentrations, in situ on lettuce and wheat seedlings	[98]
*Colletotrichum gloeosporioides* 2 strains	(*R*)-carvone and thymol (*R*)-carvone-and thymol poly(lactic acid) films	IR 77% and 100% at 20% solution	Vapor diffusion in a solid medium at 5 µL of 4 concentrations	[102]
*Aspergillus flavus*	carvone and carvone in chitosan nanoemulsion	MIC 0.8 and 0.5 μL/mL	Serial dilution in a liquid medium in situ on bread	[19]
*Aspergillus flavus*	CCEO: carvone 69.9, limonene 13.6	MIC 0.7 µL/mL	Not reported	[62]
*Aspergillus niger* *Penicillium expansum*	CCEO: carvone 54.9, limonene 43.6 and CCEO in pullulan film	MIC 0.12%, MFC 0.25% 0.12%, 0.12%	Broth dilution (CCEO), agar diffusion, and in situ on baby carrot (CCEO pullulan film)	[61]
*Aspergillus flavus* *Aspergillus luchuensis* *Aspergillus niger* *Aspergillus repens* *Aspergillus sydowii* *Penicillium purpurogenum*	CCEO: carvone 69.9, limonene 13.6 and CCEO encapsulated in chitosan	MIC 0.7 g/L and 0.25 g/L 0.6 and 0.15 g/L 0.4 and 0.18 g/L 0.3 and 0.1 g/L 0.4 and 0.12 g/L 0.5 and 0.08 g/L	Serial dilution in a liquid medium In situ on *Withania somnifera* root by fumigation	[19]
*Aspergillus flavus*	(*S*)-carvone and linalool CCEO: carvone 78.9, limonene 18.6 [2 EOs]	MIC 1500 µg/mL and >2000 µg/mL MIC 0.4%, 0.7%	Serial dilution on a solid medium	[56]
*Alternaria alternata* *Aspergillus niger* *Aspergillus ochraceus* *Aspergillus flavus* *Aspergillus terreus* *Aspergillus versicolor* *Aureobasidium pullulans* *Cladosporium cladosporioides* *Cladosporium fulvum* *Fusarium tricinctum* *Fusarium sporotrichioides* *Mucor mucedo* *Penicillium funiculosum* *Penicilium ochrochloron* *Phomopsis helianthi* *Phoma macdonaldii* *Trichoderma viride*	CCEO: carvone 46.6, limonene 45.5 [3 EOs]	MIC 0.25 µL/mL, MFC 0.25 µL/mL 1.0, 1.0 µL/mL 1.0, 2.5 µL/mL 1.0, 2.5 µL/mL 1.0, 2.5 µL/mL 0.5, 2.5 µL/mL 0.25, 0.25 µL/mL 0.25, 0.25 µL/mL 0.25, 0.25 µL/mL 1.0, 1.0 µL/mL 0.5, 1.0 µL/mL 2.5, 2.5 µL/mL 1.0, 1.0 µL/mL 1.0, 1.0 µL/mL 0.25, 0.25 µL/mL 0.25, 0.25 µL/m 10.0, 10.0 µL/mL	Microdilution in broth	[57]
*Sclerotium rolfsii*	CCEO: carvone 57.7, limonene 35.5 MSEO	MIC 2% slight activity even in 5%	Broth dilution at 4 concentrations In situ on Jerusalem artichoke	[66]
*Aspergillus flavus* 4 strains *Aspergillus parasiticus*	CCEO: carvone 72.1, limonene 23.3 [2 EOs]	MIC 1.5 µL/g, MFC 4.5 µL/g 0.7 µL/g, 1.5 µL/g	Agar dilution	[58]
*Botrytis cinerea*	CCEO: carvone main compound	IR 95% at 0.6%	Agar disk diffusion	[67]
*Aspergillus flavus* *Aspergillus ochraceus* *Aspergillus parasiticus* *Aspergillus westerdijkiae*	CCEO: carvone 52.2, limonene 41.2 [10 EOs]	MIC 125 µL/L of air 62.5 µL/L 62.5 µL/L 62.5 µL/L	Mycelial growth on solid medium in vapor phase In situ on the bread	[27]
*Fusarium solani* *Rhizoctonia solani* *Sclerotium rolfsii* *Macrophomina phaseolina*	CCEO: carvone, limonene [5 EOs]	IR 85.4% at 4% 78.8% 100% 52.2%	Mycelial growth on solid medium at 3 concentrations In situ on the bean	[60]
*Aspergillus niger*	carvone CCEO: carvone, the main compound [5 EOs, 3 compounds, 4 EO mixtures, 6 compound mixtures]	1 mg/mL 3 mg/mL	Serial broth dilution Positive control fluconazole	[68]
*Aspergillus niger* *Penicillium expansum*	CCEO: carvone 54.9, limonene 43.6 and CCEO in pullulan film	MIC 0.12%, MFC 0.25% 0.12%, 0.12%	Broth dilution (CCEO) Agar diffusion and in situ on a baby carrot	[61]
*Aspergillus flavus*	AGEO: dill apiole 33.8, carvone 27.2, limonene 13.8 and AGEO encapsulated in chitosan	MIC 1.2 µL/mL 0.6 µL/mL	Serial dilution in a liquid medium In situ on rice seeds	[37]
*Alternaria alternata* *Aspergillus flavus* *Aspergillus fumigatus* *Aspergillus niger* *Aspergillus luchuensis* *Aspergillus repens* *Aspergillus versicolor* *Cladosporium herbarum* *Fusarium oxysporum* *Fusarium poae* *Penicillium chrysogenum* *Penicillium italicum* *Penicillium spinulosum*	AGEO: dill apiole 33.8, carvone 27.2, limonene 13.8 and AGEO encapsulated in chitosan	IR 100% and 100% at 1.2 µL/mL 79 and 97% 65 and 82% 100 and 100% 100 and 100% 100 and 100% 85 and 100% 60 and 82% 100 and 100% 100 and 100% 100 and 100% 100 and 100% 70 and 100%	Mycelial growth on solid medium at the MIC dose	[37]
*Aspergillus flavus* *Aspergillus niger* *Aspergillus ochraceus* *Aspergillus terreus* *Fusarium graminearum* *Fusarium moniliforme* *Penicillium citrinum* *Penicillium viridicatum*	AGEO: carvone 55.2, limonene 16.6, dill apiole 14.4	IR 86% and 83% at 6 µL/plate 14 and 100% 19 and51% 69 and 78% 100 and 51% 9 and 62% 87 and 100% 14 and 28%	Mycelial growth inhibition by inverted Petri plate and poison food methods at 3 doses Positive control carbendazin	[65]
*Alternaria alternata* *Aspergillus flavus* *Aspergillus niger* *Aspergillus oryzae*	AGEO: carvone 41.5, limonene 32.6, apiol 16.8	2.0 µL/mL	Poisoned food technique In situ vapor phase on cherry tomatoes	[70]
*Alternaria alternata* *Bipolaris sp* *Curvularia lunata* *Fusarium oxysporum* *Stemphylium solani.*	*A. graveolens* tops and flower EO: limonene, dill apiole, carvone	IR 57.7% at 500 µg/mL/8 days 65.9 µg/mL 75.3 µg/mL 84.5 µg/mL 73.9 µg/mL	Mycelium growth on solid medium at 3 concentrations after 2–8 days	[103]
*Alternaria triticina* *Bipolaris sorokiniana*	AGEO: carvone 41.2, limonene 23.1, camphor 9.3 AGEO fractions: limonene, camphor	ED_50_ 0.38 mg/mL, ED_90_ 1.65 mg/mL 0.48, 1.3 mg/mL	Spore germination inhibition Positive control carbendazim	[31]
*Alternaria alternata* *Aspergillus flavus* *Aspergillus niger* *Aspergillus ochraceus* *Cladosporium sp.* *Fusarium oxysporum* *Penicillium expansum*	AGEO: apiol 32.8, carvone 31.0, limonene 21.3, piperitone 6.1	MIC 1/1500 (*v*/*v*) 1/370 1/150 1/370 1/370 1/370 1/370	Mycelial growth on solid medium, inhibition of sporulation and germination	[71]
*Colletotrichum nymphaeae*	AGEO: carvone 87.9, limonene 3.1 AGEO in copper nanoparticles	EC_50_ 317 mg/L 513 mg/L	Mycelium growth on solid medium at 4 concentrations and conidia germination inhibition	[96]
*Alternaria alternata* *Aspergillus flavus* *Aspergillus niger* *Colletotrichum lindemuthianum* *Penicillium sp.* *Rhizopus sp.*	AGEO: carvone 45 [13 EO:s]	IR 69% at 1000 ppm 38% 45% 25% 51% 3%	Mycelial growth inhibition on solid medium	[72]
*Penicillium digitatum*	AGEO: carvone 39.4, limonene 33.5 [15 EOs]	MIC 5 µL/mL, 5 EC_50_ 1.6 µL/mL	Serial dilution in a solid medium In situ on citrus fruit	[73]
*Lecanicillium fungicola var. fungicola*	AGEO: carvone 33.2, limonene 19.9, dill apiole 17.6 [11 EOs]	EC_50_ 408.4 µL/L MIC 750–1000 µL/L	Mycelium growth on solid medium at serial dilution	[59]
*Aspergillus niger* *Aspergillus flavus*	(+)- and (−)-carvone/(+) and ASEO and MSEO AGEO: carvone 50.4, limonene 21.4, dill apiole 17.7 MSEO: carvone 56.6, limonene 27.3	IZ 17 and 12 and 7 and 6 mm IZ 9 and 5 mm	Agar disk diffusion (5 µL) and broth serial dilution	[104]
*Aspergillus flavus*	AGEO *A. graveolens* leaf oil	MIC 1.25 µL/mL 7.0 µL/mL	Poisoned food technique	[74]
*Absidia ramosa* *Alternaria alternata, Aspergillus flavus, Aspergillus fumigatus, Aspergillus glaucus* *Aspergillus luchuensis* *Aspergillus niger* *Aspergillus terreus* *Aspergillus unguis, Cladosporium cladosporioides, Curvularia lunata, Fusarium oxysporum Mucor sp., Mycelia sterilia, Penicillium citrinum, Penicillium italicum, Penicillium luteum, Penicillium purpurogenum, Rhizopus stolonifer, Spondylocladium australe*	MSEO: carvone 59.6, limonene 25.6	100% at 1.0 µL/mL 100 91.7 100 75.7 100	Serial dilution in a liquid medium Aflatoxin B1 inhibition Positive control Nystatin and Wettasul-80	[94]
*Aspergillus flavus*	*Mentha cardiaca*: carvone 59.6, limonene 23.3	MIC 1.25 µL/mL, MFC 2.25 µL/mL	Mycelial growth inhibition on solid medium	[76]
*Aspergillus flavus* *Aspergillus fumigatus* *Aspergillus niger* *Aspergillus sydowii* *Aspergillus sulphureus* *Aspergillus terreus* *Fusarium sp.* *Penicillium purpurogenum* *Rhizopus stolonifer*	*Mentha cardiaca*: carvone 59.6, limonene 23.3	IR 100% at 1.25 µL/mL 55% 95% 100% 100% 50% 100% 100% 0%	Mycelial growth inhibition on solid medium	[76]
*Aspergillus niger* *Botryodiplodia theobromae* *Fusarium solani* *Mucor mucedo* *Rhizopus solani*	MSEO/carvone/cis-carveol MSEO: carvone 51.7, cis-carveol 24.3, limonene 5.3	MIC 0.07/0.03/0.03 mg/mL 0.11/0.10/0.09 mg/mL 0.09/0.11/0.11 mg/mL 0.07/0.08/0.09 mg/mL 0.09/0.10/0.15 mg/mL	Broth microdilution and disk diffusion positive test for flumequine	[77]
*Alternaria alternata* *Alternaria solani* *Aspergillus niger* *Aspergillus flavus* *Fusarium solani* *Rhizopus solani* *Rhizopus spp.*	MSEO summer: carvone 59.5, limonene 10.5 MSEO winter: carvone 63.2, limonene 9.1 [4 mint EOs, 4 compounds]	MIC 122 µg/mL, 133 µg/mL 81 and 101 µg/mL 89 and 107 µg/mL 81 and 69 µg/mL 90 and 92 µg/mL 53 and 59 µg/mL 103 and 130 µg/mL		[77]
*Aspergillus niger*	MSEO: carvone 57.7–65.6, germacrene D 9.4–12, limonene 4.2–9.6 *Mentha longifolia* EO: carvone 64.7–72, limonene 7.2–12.9 [4 mint EOs at 3 stages]	MIC 30 µg/mL, MFC 60 µg/mL 30 µg/mL/60 µg/mL	Broth microdilution Disk diffusion method, 15 µL/disk Positive control Amphotericin-B	[105]
*Aspergillus flavus* *Aspergillus fumigatus* *Aspergillus niger*	MSEO: carvone 62.9, limonene 8.5 [2 mint EOs]	MIC 1.25 µL/mL, MFC 5 µL/mL 0.64, 2.5 µL/mL 0.64, 2.5 µL/mL	Broth microdilution	[106]
*Aspergillus flavus* *Aspergillus niger* *Penicillium stoloniferum*	MSEO: carvone 58.7, limonene 10.7 [4 mint EOs]	MIC 1.0, MFC 1.0 mg/mL 1.0, 1.0 mg/mL 2.0, 2.0 mg/mL	Serial dilution in solid medium, MIC/MFC Disk diffusion method, 10 µL/disk Positive control benomyl	[107]
*Trichoderma harzianum* *Verticillium fungicola*	MSEO: carvone 49.5, menthone 21.9, limonene 5.8 [10 EOs, 10 compounds]	MIC 1.0–2.5 and 3.5–5 µL/mL 2 and 5 µL/mL MIC 0.5–1.5, MFC 5 µL/mL	Broth microdilution and macrodilution in solid medium, vapor test, positive control bifonazole	[79]
*Alternaria alternata*, *Aspergillus flavus*, *Aspergillus niger*, *Aspergillus ochraceus*, *Aspergillus terreus*, *Aspergillus versicolor*, *Cladosporium cladosporioides*, *Penicillium funiculosum*, *Phomopsis helianthi*, *Penicillium ochrochloron*, *Trichoderma viride*, *Fusarium tricinctum*, *Phomopsis helianthin*, *Trichoderma viride*	carvone MSEO: carvone 49.5, menthone 21.9, limonene 5.8 [4 EOs, 4 compounds]	MIC 0.25–1.0 µL/mL 1.0–2.5 µL/mL	Serial broth microdilution and macrodilution on solid medium, positive control bifonazole	[81]
*Alternaria* sp. *Aspergillus flavus* *Aspergillus niger* *Aspergillus ochraceus* *Aspergillus parasiticus* *Fusarium culmorum* *Fusarium graminearum* *Fusarium moniliforme* *Fusarium oxysporum* *Penicillium citrinum* *Penicillium expansum* *Penicillium viridicatum* *Pyrenophora graminea*	carvone 52.2, limonene 21.6 MSEO: carvone 55 [3 EOs]	MIC 10 µL/mL, MFC 10 µL/mL 2.5, 5 5, 10 2.5, 5 5, 10 5, 10 2.5, 2.5 2.5, 2.5 2.5, 2.5 5, 10 5, 10 5, 10 5, 10	Broth microdilution	[63]
*Aspergillus terreus* *Fusarium oxysporum* *Penicillium expansum* *Verticillium dahliae*	carvone and MSEO: carvone 55 [4 EOs, 6 compounds]	Growth 0% and 0% at 10 µL/disk 54 and 90% 0 and 0% 11 and 0%	Disk diffusion at 3 doses, mycelial growth in respect to the control	[108]
*Verticillium dahliae*	MSEO: carvone 42.0, trans-carveol 15.6, dihydricarvyl acetate 12.7, neodihydrocarveol 12.5 [6 EOs]	IR 100% at 16 µL/dish	Vapor test on a Petri dish	[82]
*Rhizopus stolonifer*	MSEO: carvone 51.3, limonene 21.3 [23 EOs]	IR 92% at 150 μL/L	Vapor test on a solid medium In situ on strawberry and peach fruits	[83]
*Geotrichum citri-aurantii*	(*R*)-carvone (*S*)-carvone MSEO: carvone 87.9, limonene 2.6 [59 EOs, 10 compounds]	IR 100% at 1000 µL/L 69% 31%	Mycelial growth inhibition on solid medium at 500 and 1000 µL/L Positive control 5 fungicides	[78]
*Lasiodiplodia theobromae* *Fusarium pallidoroseum* *Fusarium solani*	(*R*)-carvone, (*S*)-carvone *Lippia alba* EO: 2 samples: carvone 53.0, 63.5, limonene 27.0, 25.9 [2 EOs]	MIC 0.1/0.2/0.2./0.2 mL/100 mL 0.2/0.2/0.2./0.2 mL/100 mL 0.1/0.1/0.2/0.2 mL/100 mL	Serial dilution on a solid medium	[85]
*Botryosphaeria parva* *Colletotrichum gloeosporioides*	(*R*)-carvone, *(S*)-carvone *Lippia scaberrima* EO: limonene 38, carvone 34, 1,8-cineole 5	MIC 1600/1600/2400 µL/L MIC 1600/1600/>2400 µL/L 2000 µL/L	Serial dilution on a solid medium and vapor test on Petri dishes In situ on avocado	[86]
*Alternaria* sp. *Colletotrichum gloeosporioides* *Lasiodiplodia theobromae*	(*R*)-carvone (*S*)-carvone *Lippia scaberrima* EO: limonene 38, carvone 34, 1,8-cineole 5	MIC 1000/1000/2000 µL/L 1000/1000/1000 µL/L 1000/1000/2500 µL/L	Serial dilution on a solid medium and vapor test on Petri dishes In situ on mango	[87]
*Botrytis cinerea*	*Aloysia polystachya* EO: carvone 85.9, limonene 9.4	MIC 71 µL/L air (mycelial growth) 47.2 µL/L air (conidial germination)	Vapor test at serial dilutions In situ on cherry tomatoes	[88]
*Penicillium digitatum*	(*R*)-carvone MSEO: composition not reported *Lippia scaberrima* composition not reported	MIC 1000 µL/L 1000 µL/µL/L 3000 µL/L	Serial dilution on a solid medium In situ on citrus fruit	[89]
*Alternaia alternata* (1 strain) *Alternaria citrii* (1strain) *Botrytis cinerea* (1strain) *Colletotrichum gloeosporioides* (2 strains) *Lasiodiplodia theobromae* (2 strains) *Penicillium digitatum* (3 strains)	(*R*)-carvone, (*S*)-carvone CCEO: (*S*)-carvone 67.6, limonene 22.8 AGEO: (*S*)-carvone 57.9, limonene 21.9 MSEO: (*R*)-carvone 87.9, limonene 2.6 *Lippia scaberrima* EO: (*R*)-carvone 49.3, limonene 20.2 [18 EOs, 7 compounds]	MIC 1000–3000/1000–3000 µL/L 2000–>3000 µL/L 2000–>3000 µL/L 2000–3000 µL/L 1000–3000 µL/L	Serial dilution on a solid medium, mycelial growth inhibition Thyme oil and thymol were the most active, followed by eugenol-rich and carvone-rich EOs	[90]

MIC—minimal inhibitory concentration; MFC—minimal fungicidal concentration; MQ—minimal quantity that caused an apparent inhibition zone; IR—inhibition rate, no data given.

**Table 2:** In vitro antibacterial activity of carvone and carvone-rich essential oils against phytopathogens.

Bacteria	Compound EO: Main Compounds [%] [No. of EOs/Compounds Tested]	Results	Methods	Ref.
*Xanthomonas campestris, Erwinia carotovora*	(−)-carvone and (−)-carvone-PLGA-composite	400 μg/mL and 150 μg/mL	Positive control gentamycin and chloramphenicol	[98]
*Agrobacterium tumefaciens* *Clavibacter michiganensis* (2 ssp.) *Curtobacterium flaccumfacien* *Erwinia carotovora* (2 subsp.) *Pseudomonas syringae* (10 pv.) *Pseudomonas tolaasii* *Pseudomonas* (5 sp.) *Ralstonia solanacearum* *Rhodococcus fascians* *Xanthomonas campestris* (4 pv.)	CCEO: carvone 23.3, limonene 18.2, germacrene D 16.2, trans-dihydrocarvone 14.0 [2 EOs]	MQ 682 µg/disk; 690 µg/disk 256 and 455 µg/disk 455 and 628 µg/disk 455 and 910 µg/disk 910–5460 and 920–7360 µg/disk 910 and 920 µg/disk 910–7280 and 920–7360 228 and 230 µg/disk 910 and 460 µg/disk 170–455 and 345–920 µg/disk	Agar diffusion at serial dilution Positive control rifampicin	[25]
*Xanthomonas campestris*	(*R*)-carvone (*R*/*S*)-limonene CCEO: carvone 58.4, limonene 31.1	MIC 125 μg/mL, MBC 1000 μg/mL >1000 μg/mL 125, 500 μg/mL	Broth dilution Positive control seven antibiotics	[69]
*Agrobacterium tumefaciens* *Pseudomonas syringae* *Xanthomonas arboricola*	MSEO: carvone 73.4, limonene 3.5 *Mentha* × *rotundifolia* EO: carvone 51.5, limonene 5.9 [6 mint EOs]	IZ 14.7 mm and 14.1 mm 10.8 and 10.3 mm 14.5 and 12.9 mm	Disk diffusion, 10 µL Positive control streptomycin	[109]
*Pseudomonas tolaasii*	MSEO: carvone 49.5, menthone 21.9, limonene 5.8 [10 EOs, 10 compounds]	MIC 1.0–2.5 and 3.5–5 µL/mL 2 and 5 µL/mL MIC 0.5–1.5, MFC 5 µL/mL	Disk diffusion and broth microdilution, positive control streptomycin and penicillin	[79]

MIC—minimal inhibitory concentration; MFC—minimal fungicidal concentration; MQ—minimal quantity that caused an apparent inhibition zone; IR—inhibition rate, no data given.

### 4.2. Biological Effects on Arthropod Pests

Arthropods make up over 80% of animal species [110]. Some, like insects and mites, are major agricultural pests, causing crop losses and food contamination. Controlling these pests is vital in agriculture, medicine, and veterinary science.

#### 4.2.1. Insecticidal Effects

Data on the insecticidal activity of EOs derived from *C. carvi*, *A. graveolens*, and *M. spicata* are presented in Table 3. EOs have demonstrated significant insecticidal properties against a wide range of insect orders, including Coleoptera, Diptera, Hemiptera, Isoptera, and Lepidoptera [7,111]. The target species include major agricultural pests, such as aphids, beetles, flies, moths, termites, thrips, weevils, and whiteflies. Treatment with CCEO, AGEO, and MSEO has been shown to induce acute lethal effects on insect pests. The lethality of these pests was assessed using three bioassay methods: contact toxicity, fumigant toxicity, and oral toxicity (Table 3). These findings underscore the potential of these EOs for managing insect pests in both outdoor and indoor environments, including fields, orchards, storage facilities, greenhouses, and even residential areas. Moreover, the toxicity of these EOs has been demonstrated across various life stages of insect pests. For instance, fumigation with MSEO at a concentration of 0.1 μL/mL resulted in a 100% ovicidal effect [99], 88.8% larvicidal activity, and 72.9% pupicidal activity against *C. chinensis* [64].

In addition to acute lethality, numerous sublethal and chronic effects of carvone-rich EOs on insect pests have been reported. The repellent effect is one of the well-documented sublethal effects of carvone-rich EOs. For example, Kłyś et al. [112] reported that CCEO and its main compound, carvone, exhibit considerable toxicity and adult repellency against *S. oryzae*. In the study of Girardi et al. [113], the repellent activity of EOs isolated from three different accessions of *C. carvi* was evaluated against the green peach aphids *Myzus persicae* (Sulzer). It was found that the repellent activity of CCEO depends on the proportions of carvone and limonene. Specifically, EO with a similar limonene-to-carvone ratio (53% and 46%, respectively) demonstrated higher and more stable in-time repellency. More examples of the repellency of CCEO, AGEO, and MSEO against insect pests are presented in Table 3. The repellent effects of insecticides play a significant role in insect pest management, as they prevent insects from being attracted to host or protected areas, thereby reducing pest populations and the damage they cause. These effects can reduce the need for frequent insecticide use and the development of insecticide resistance.

Feeding deterrence is one of the different sublethal effects of EOs. For example, treatment of *C. chinensis* adults with MSEO at 0.1 μL/mL resulted in 100% mortality of adults and eggs, complete repellency, and a 100% feeding deterrence index [64]. According to Dancewicz et al. [114], CCEO has a significant feeding deterrent effect on *M. persicae*, which is attributed to its main component, carvone. Rosa et al. [115] reported that AGEO and its main compound, carvone, exhibit considerable insecticidal effects, including acute contact toxicity, fumigant toxicity, and repellency, and are antinutritional to adults of *S*. *zeamais*. According to Kostić et al. [116], the nutritional indices of fourth instar larvae of *L. dispar*, including AD (approximate digestibility), ECD (efficiency of conversion of digested food), ECI (efficiency of conversion of ingested food), RCR (relative consumption rate), RGR (relative growth rate), and RMR (relative metabolic rate), were reduced by AGEO. The compounds in EOs likely disrupt the signaling pathways that stimulate feeding in the pest. More examples of EOs’ potential as feeding deterrents are presented in Table 3.

The inhibitory effects of EOs on detoxification enzymes and the antioxidative defense system of insect pests have also been proven. For example, according to Petrović et al. [117], the decreased activities of catalase, superoxide dismutase, and glutathione-S-transferase in *T. confusum* and *T. molitor* adults exposed to CCEO indicate that, in addition to its direct fumigant action, the oil also provokes significant oxidative stress. In the other study, the use of AGEO (LC_50_ 17.32 μg/μL) significantly reduced the activity of detoxification enzymes, including acetylcholinesterase, α- and β-carboxylesterase, and glutathione S-transferase, in *R. dominica* adults [118]. In a similar study with AGEO, diverse insecticidal effects, including lethal contact toxicity, fumigant toxicity, repellency, oviposition inhibition, and reduction in acetylcholinesterase activity, were reported [119]. The same results on the acetylcholinesterase inhibitory effects of MSEO and carvone against *R. dabieshanensis* were also reported [120]. Given that detoxifying enzymes play a significant role in the development of resistance in insect pests to insecticides [121], inhibiting their activity through plant EOs can reduce the likelihood of resistance emerging in pest insects.

Compatibility with other insecticidal agents is another advantage of using EOs and carvone in insect pest management. For example, Yoon et al. [122] demonstrated a synergistic repellent effect between carvone and limonene against adults of *S. oryzae*. Their study found that carvone alone was strongly repellent to the pest at 6 and 12 µL after 24 h, while limonene was effective only at 8 µL, showing no activity at 4 µL. However, the equal combination of the compounds resulted in significantly enhanced efficacy, achieving repellency rates of 93.3% and 96.7% at 10 µL and 20 µL, respectively. You et al. [123] found that (*R*)-carvone synergizes with perillaldehyde toxicity in *Tribolium castaneum*, linking this effect to a suppression of detoxification and elimination mechanisms. In an evaluation of the synergistic effects of 12 monoterpenoids against *Poratrioza sinica* (Hemiptera: Psyllidae) by Yang et al., (*R*)-carvone was identified as the most effective synergist. Furthermore, its binary mixtures with dihydrocarvone, cuminaldehyde, cuminyl alcohol, (*S*)-carvone, and estragole showed significant potential as control agents for this pest [120]. In another study, Jayaram et al. [124] reported that MSEO has synergistic effects on the toxicity of the EO of *Tagetes minuta* L. against adults of the pulse beetles, *C*. *chinensis* and *C*. *maculatus*. The 24 h-LC_50_ values of *T. minuta* EO against these pests were 3.5 and 3.4 µL/mL, respectively, and were reduced to 1.5 and 2.4 µL/mL by combination with MSEO. The compatibility of such agents will enable their combined application in pest management.

A recent study by Mondal et al. [125] demonstrated that the nanoemulsification of MSEO and its main constituent carvone (81.9%) is a novel and effective method for developing potent aphicides against the corn aphid, *Rhopalosiphum maidis* (L.), and the wheat aphid, *Sitobion avenae* (F.). It was found that the nanoemulsion and carvone had significant toxicity (24 h-LC_50_ values of 2.87–2.81 and 0.87–1.94 mg/mL, respectively) and acetylcholinesterase inhibitory effects (IC_50_ values of 1.66–5.34 and 0.07–3.83 mg/mL, respectively) against both aphids. It was found that MSEO-loaded chitosan nanoparticles have strong insecticidal efficacy against adults of *C. maculates* (LC_50_ = 56 μL/L) and *S. granarius* (LC_50_ = 47 μL/L) [126]. In the other study, Prabhakar et al. [127] evidenced the significant insecticidal potential of the *Ocimum gratissimum* L. The EO (thujone 29.4%) and MSEO (carvone 59.0%) combination (1:1 ratio) was incorporated into the polylactic acid polymer matrix as a composite against *S. oryzae* and the sawtoothed grain beetle (*Oryzaephilus surinamensis* (L.)) in sorghum and pearl millet. Therefore, novel formulations, including nanoemulsions and polymeric composites, represent a highly effective strategy for deploying carvone-rich EOs in insect pest management.

**Table 3:** Insecticidal effects of carvone-rich essential oils.

Insect(s) and Stage	EO: Main Components [%]	Insecticidal Effects	Methods	Ref.
*Sitophilus oryzae*, adults	CCEO: Carvone 61.9, limonene 38.1	RI is 91.7% at 10 µL after 24 h	Repellent effect using a T-tube olfactometer	[122]
*Lycoriella ingénue*, larvae	CCEO: Carvone 53.7, limonene 43.4	100% mortality at a concentration of 0.005 mg/mL air after 24 h	FT using treated filter papers in a glass cylinder	[128]
*Sitophilus zeamais*, adults *Tribolium castaneum*, adults	CCEO: Carvone 38.0, limonene 26.6, α-pinene 5.2, carveol 5.0, β-myrcene 4.7	CT: 24 h-LD_50_ values are 3.07 and 3.29 μg/adult, respectively FT: 24 h-LC_50_ values are 3.37 and 2.53 mg/L, respectively	CT by topically treating the dorsal thorax of the pest, FT using treated filter papers in a glass vial	[129]
*Brassicogethes aeneus*, adults	CCEO: Carvone 61.5, limonene 32.7, myrcene 3.2	CT: 24 h-LC_50_ is 126.0 μg/cm^2^ RI is 79.2% at a concentration of 10 μL/mL after 24 h	CT using the arsal test Repellent effect using treated flowering sprouts of oilseed rape	[130]
*Sitophilus oryzae*, adults	CCEO: Carvone 40.8, limonene 27.1	48 h-LC_50_ value is 2.5 mg/L	FT using treated filter paper in a glass cylinder	[131]
*Spodoptera littoralis*, larvae	CCEO: Carvone 67.6, limonene 28.5	24 h-LC_50_ is 41.451 μL/L	FT using treated filter papers in the cup as a fumigation chamber	[132]
*Tenebrio molitor*, adults *Tribolium confusum*, adults	CCEO: Carvone (68.22 ± 0.62), limonene (21.80 ± 0.54)	48 h-LC_50_ values are 3.3 and 4.0 μL/mL, respectively	FT using treated filter papers in a glass cylinder	[117]
*Callosobruchus maculatus*, adult females and males	CCEO: Carvone 46.2, *ρ*-cymene 14.0, γ-terpinene 8.3, carvone oxide 6.2	FT: 24 h-LC_50_ values are 2.99 and 0.42 μL/L, respectively Repellency: RP is 66.3 after 30 min	FT using a cylindrical container Repellent effect using a Y-tube olfactometer	[133]
*Myzus persicae*, apterous adults	CCEO: Carvone 47.3–74.4, limonene 25.2–51.9	RI is 32% and 41% at 90 and 330 min, respectively	Repellent effect using a dual-choice bioassay with white cabbage	[113]
*Lasioderma serricorne*, adults, *Sitophilus oryzae*, adults, *Tribolium castaneum*, adults	CCEO: Carvone 72.4, limonene 23.9	24 h-LC_50_ values are 28.0, 118.4, and 1.9.34 µL/L, respectively	FT using treated filter papers in Plexiglas bottles	[134]
*Sitophilus oryzae*, adults	AGEO: Carvone 40.8, limonene 22.8, dill ether 5.0, α-phellandrene 3.9, *p*-cymene 3.1	48 h-LC_50_ is 3.3 mg/L	FT using treated filter papers in a glass cylinder	[131]
*Mythimna unipuncta*, larvae	AGEO: Carvone 84, β-phellandrene 14 *A. graveolens* infrutescences EO: Carvone 67, β-phellandrene 25	FDI (17.7 and 84.7%, respectively) and growth inhibition (32.5 and 81.2%, respectively) at 175 μg/cm^2^ after 48 h CT (88.3 and 100% mortality at 250 μg/cm^2^ after 24 h, respectively) and FT (90.0 and 100% mortality at 250 μg/cm^3^ after 24 h, respectively)	Feeding deterrence and growth inhibition using the diet-no-choice method with corn leaves as food, CT using treated filter papers in Petri dishes, FT using treated filter papers in fumigation jars	[135]
*Mythimna unipuncta*, eggs	*A. graveolens* infrutescences EO: Carvone 66.6, β-phellandrene 24.7	100% after 6 days at a concentration of 15 mg/mL, along with increased lifespan and decreased pupal weight	Egg hatching is inhibited by dipping in freshly prepared solutions of EO	[136]
*Ephestia kuehniella*, larvae	*A. graveolens* herb EO: Phellandrene 34.2, carvone 23.7, limonene 21.5, α-terpineol 5.6, *p*-cymene 5.5	24 h-LC_50_ is 18.0 µL/L	FT using treated filter papers in glass jars	[137]
*Rhyzopertha dominica*, adults	AGEO: Carvone 30.2, dihydrocarvone 22.9	100% mortality at a concentration of 40 μL/L after 24 h, along with enzymatic disruption; reduction in the activity of acetylcholinesterase, α- and β-carboxylesterase, and glutathione-s-transferase	FT using treated filter papers in glass vials	[118]
*Tribolium castaneum*, adults	AGEO: Dillapiole 37.9, carvone 22.6, isolimonene 10.0, dihydrocarvone 6.9, camphor 5.1, α-phellandrene 2.8	FT: 24 h-LC_50_ is 132.6 μL/L air RC_50_ = 0.01 μL/cm^2^ after 1 h	FT using treated filter papers in a cylindrical vial, repellent effect using the preferential zone method on filter paper	[138]
*Rhyzopertha dominica*, adults	MSEO: Carvone 79.9, 1,8 cineole 4.3	100% mortality at the dose of 3.12% after 24 h	CT using treated filter papers	[139]
*Lycoriella ingénue*, larvae	MSEO: Carvone 63.2, limonene 19.4, 1,8-cineole 1.8	100% mortality of larvae at a concentration of 0.005 mg/mL air after 24 h	FT using treated filter papers in a glass cylinder	[128]
*Sitophilus granaries*, adults	MSEO: Carvone 59.4, ocimene 6.7, 1,8-cineole 2.1	24 h-LC_50_ is 0.08 μL/mL	FT using treated filter papers in a glass jar	[140]
*Callosobruchus chinensis*, eggs, larvae, pupae, and adults	MSEO: Carvone 59.6, limonene 25.6, *p*-cymene 2.8, 1,8-cineole 2.5	98.5% oviposition deterrence of adult females along with 100% ovicidal and larvicidal, 72.9% pupicidal, and 100% FDI at 0.1 μL/mL; 100% repellency of adults at 0.03 μL/mL after 30 min	FT using treated filter papers in a plastic jar, repellent effect using the Y-shaped olfactometer	[94]
*Ephestia kuehniella*, eggs, larvae, pupae, and adults *Plodia interpunctella*, eggs, larvae, pupae, and adults	MSEO: Carvone 67.1, limonene 14.3, γ-muurolene 2.3, myrcene 2.1	24 h-LC_50_ values of 896.5, 2277.6, 1824.3, and 0.5 μL/L for *E. kuehniella* and 1231.4, 2437.5, 1981.9, and 0.4 μL/L for *P. interpunctella*, respectively	FT using treated filter papers in glass jars	[141]
*Rhyzopertha dominica*, adults	MSEO: Carvone 48.5, limonene 20.7, 1,8-cineole 5.4	43% mortality at a concentration of 2 μL/mL after 96 h 56.2% repellency after 30 min at μL/mL	FT by the treatment of a bit of cotton in glass jars, repellent effect using treated filter papers in a Petri dish	[142]
*Bactrocera oleae*, adults	MSEO: Carvone 54.1, limonene 21.9	40.3% mortality at 15 μL/L after 24 h	FT using treated filter papers in plastic jars	[143]
*Myzus persicae*, apterous adults, *Rhopalosiphum padi*, apterous adults, *Spodoptera littoralis*, larvae	MSEO: Carvone 79.0, 1,8-cineole 12.0, menthol 2.0	Feeding inhibitory against *S. littoralis* (72.8%) and setting inhibition against *M. persicae* and *R. padi* (57.7 and 17.5%, respectively) at 100 μg/cm^2^.	Feeding/settling on the treated leaf disks	[144]
*Reticulitermes dabieshanensis*, adult workers	MSEO: Carvone 52.3, limonene 19.8, dihydrocarvone 11.1	24 h-LC_50_ is 0.19 μL/L, along with a significant acetylcholinesterase inhibitory effect	FT using treated filter papers in glass jars	[120]
*Callosobruchus chinensis*, adults *Callosobruchus maculatus*, adults	MSEO: Carvone 63.4, limonene 21.3, 1,8-cineole 2.3.	FT: 24 h-LC_50_ are 1.9 and 2.4 μL/mL, respectively RI = 0.92 and 0.96 after 4 h, respectively, indicating significant repellency; ovipositional deterrence: 100% at 10 μL/mL and 94.26% 12 μL/mL after 24 h, respectively	FT using filter papers in glass desiccators Repellent effect using treated filter papers in Petri dishes	[124]

Scientific and common names of insect pests: *Bactrocera oleae* (Rossi) (Diptera: Tephritidae) (the olive fruit fly); *Callosobruchus chinensis* L. (Coleoptera: Chrysomelidae) (the pulse beetle); *Callosobruchus maculatus* (F.) (Coleoptera: Chrysomelidae) (the cowpea weevil); *Ephestia kuehniella* Zeller (Lepidoptera: Pyralidae) (the Mediterranean flour moth); *Lasioderma serricorne* F. (Coleoptera: Anobiidae) (the cigarette beetle); *Lycoriella ingenua* (Dufour) (Diptera: Sciaridae)) (the mushroom fly); *Lymantria dispar* L. (Lepidoptera: Erebidae) (the gypsy moth); *Meligethes aeneus* (F.) (Coleoptera: Nitidulidae) (the pollen beetle); *Myzus persicae* (Sulzer) (Hemiptera: Aphididae) (the green peach aphid); *Plodia interpunctella* (Hubner) (Lepidoptera: Pyralidae) (the Indian meal moth); *Pseudaletia unipuncta* (Haworth) (Lepidoptera: Noctuidae) (true armyworm); *Reticulitermes dabieshanensis* (Isoptera: Rhinotermitidae) (the subterranean termite); *Reticulitermes speratus* Kolbe (Isoptera: Rhinotermitidae) (the Japanese termite); *Rhopalosiphum padi* L. (Hemiptera: Aphididae) (the bird cherry-oat aphid); *Rhyzopertha dominica* (F.) (Coleoptera: Bostrichidae) (the lesser grain borer); *Sitophilus granarius* L. (Coleoptera: Curculionidae) (the granary weevil); *Sitophilus oryzae* (L.) (Coleoptera: Curculionidae) (the rice weevil); *Sitophilus zeamais* Motschulsky (Coleoptera: Curculionidae) (the maize weevil); *Spodoptera littoralis* Biosduval (Lepidoptera: Noctuidae) (African cotton leafworm); *Tenebrio molitor* L. (Coleoptera: Tenebrionidae) (the yellow mealworm beetle); *Tribolium castaneum* Herbst (Coleoptera: Tenebrionidae) (the red flour beetle); *Tribolium confusum* du Val (Coleoptera: Tenebrionidae) (the confused beetle). Other abbreviations: CT: Contact toxicity; EC_50_ = 50% Effective concentration (death/inhibitory/immotility); FDI: Feeding deterrence inhibitory; FT: Fumigant toxicity; LC_50_: Concentration to kill 50% of tested organism; LD_50_: Dose to kill 50% of tested organism; RI: Repellency index; RC_50_ = 50% repellent concentration.

#### 4.2.2. Acaricidal Effects

The acaricidal activity of carvone-rich EOs isolated from *C. carvi* and *M. spicata* has been documented in recent studies. Spider mites (Trombidiformes: Tetranychidae) are among the most important pests of agricultural, horticultural, and ornamental plants worldwide due to their ability to produce silk, wide host range, high reproductive capacity, and resistance to numerous pesticides [145,146]. Recent studies indicate that EOs have high potential for managing spider mites [147,148,149,150]. For example, CCEO and MSEO were toxic to the chlorpenapyr (24 h-LC_50_ = 34.4, 12.1, and 42.2 μg/cm^3^, respectively), fenpropathrin (24 h-LC_50_ = 46.2, 18.5, and 44.8 μg/cm^3^, respectively), pyridaben (24 h-LC_50_ = 44.1, 16.6 and 44.5 μg/cm^3^, respectively), and abamectin-resistant (24 h-LC_50_ = 48.0, 20.3, and 49.0 μg/cm^3^, respectively) two-spotted spider mite, *Tetranychus urticae* Koch [151]. Furthermore, it was indicated that the predator mite *Neoseiulus californicus* McGregor was 1–2 times more tolerant than *T*. *urticae* to EOs. Yu et al. [152] reported that CCEO at 1000 ppm, containing 73.3% carvone, repels 92.2% of *T. urticae* adults. In another study with CCEO rich in carvone (66.7%), significant contact toxicity to adult *T. urticae* was reported, with a 24 h-LC50 of 13,437.8 ppm [153]. In the study by Sertkaya et al. [154], MSEO, dominated by carvone (59.4%), exhibited promising fumigant toxicity against adults of the carmine spider mite, *Tetranychus cinnabarinus* (Boisd.), with a 24 h-LC_50_ of 1.8 μg/mL. At a higher concentration of 10.0 μg/mL, MSEO achieved 100% mortality of the pest. MSEO was also toxic to the eggs and adults of *T. urticae* with 24 h-LC_50_ values of 19.7 and 21.0 μL/L, respectively [155]. The carvone was also dominant in this EO (55.0%). Kheradmand et al. [156] demonstrated that MSEO, which contains high levels of carvone (59.4%), has significant fumigant toxicity against both eggs and adults of *T. urticae*, reporting LC_50_ values of 9.0 and 7.5 μL/L, respectively. Furthermore, a concentration of 4.5 μL/L significantly repelled the adults, with a mean repellence index of 0.76 after 24 h.

The varroa mite (*Varroa destructor* (Anderson and Trueman) (Mesostigmata: Varroidae) is one of the most important pests of honeybees worldwide, attacking larvae, pupae, and adults in colonies, causing extensive economic damage [157]. It was found that the CCEO can be used to treat honeybees infected with the varroa mite, as a safe way to control the mite and protect the honeybees. According to [158], the carvone-rich CCEO (35.4%) can significantly reduce varroa mite infestation levels on adult and brood workers, with no statistical difference compared with the conventional synthetic acaricide Apistan. Furthermore, bioassays performed on worker honeybees as a biomarker of DNA damage indicated a significant increase in DNA damage with Apistan (20.1%) and infested bees with varroa mites (21.6%) compared with the corresponding treatments with CCEO (12.4%). A concentration of 1% AGEO and MSEO caused mortality of *V. destructor* within three (98.3 and 82.5%, respectively), which was significantly more toxic than the other eight tested EOs [159]. Accordingly, these EOs can be considered eco-friendly and safe agents for the management of the varroa mite.

In addition to their acaricidal potential against agricultural pests, the EOs show promising efficacy against veterinary and human ticks. For example, the lone star tick (*Amblyomma americanum* (L.) (Ixodida: Ixodidae) was repelled by AGEO with 43.2% carvone [160]. This mite can transmit dangerous diseases such as Ehrlichiosis and Tularemia to humans, and Cytauxzoonosis to animals. The susceptibility of the Mediterranean Hyalomma tick (*Hyalomma lusitanicum* Koch (Ixodida: Ixodidae)) and the sheep tick (*Ixodes ricinus* (L.) (Ixodida: Ixodidae)) to carvone-rich MSEO was also reported [144,161]. The use of EOs to control such mites as a healthy and aromatic agent also appears very promising.

#### 4.2.3. Relation of Insecticidal and Acaricidal Effects with Chemical Composition

A survey of the literature indicates a relationship between carvone and other terpenes and the insecticidal activity of EOs. For instance, López et al. [162] demonstrated that the toxicity of CCEO against *S. oryzae* adults was linked to its chemical profile, specifically its high carvone content. In the study by Seo et al. [163], carvone (48.7%) and limonene (24.2%) in the CCEO and carvone (35.6%), limonene (20.5%), and α-phellandrene (4.9%) in the AGEO were the dominant compounds. They found that all compounds had fumigant toxicity against Japanese termite *R. speratus*, with carvone showing the greatest toxicity (100% mortality at 0.25 mg/filter paper after 7 days). They also concluded that the insecticidal effects of CCEO and AGEO were related to their pure components, such as carvone. Fang et al. [129] concluded that the insecticidal effects of CCEO against *S. zeamais* and *T. castaneum* adults were related to its chemical composition, particularly the (*R*)-carvone and limonene content, as evidenced by the strong contact and fumigant toxicity. It was also indicated that the carvone-rich MSEO (52.3%) had significant fumigant toxicity and acetylcholinesterase inhibitory activity on adult workers of *R. dabieshanensis*. Similarly, carvone and the other dominant terpene, limonene, showed considerable fumigant toxicity and acetylcholinesterase inhibitory activity. Furthermore, it was found that carvone, with an IC_50_ value of 2.4 μL/mL, was the most effective compound and that, in a binary treatment with limonene for acetylcholinesterase inhibition, a synergistic effect was observed [120]. It was concluded that the insecticidal efficacy of MSEO was due to its active ingredient, particularly carvone.

Different parts of a plant selected for EO isolation can exhibit diverse pesticidal effects, which can be attributed to variations in their chemical profiles. According to the study by Sousa et al. [135], the EOs isolated from green infrutescences and mature fruits of *A. graveolens* exhibit distinct chemical profiles, with carvone (67 and 84%, respectively) and β-phellandrene (25 and 14%, respectively) as the dominant components. Furthermore, the insecticidal effects from contact (88.3 and 100% mortality, respectively, at 250 μg/cm^2^ after 24 h) and fumigant (90.0 and 100% mortality at 250 μg/cm^3^, respectively, after 24 h) toxicity to feeding deterrence (17.7 and 84.7%, respectively, at 175 μg/cm^2^ after 48 h) and growth inhibition (32.5 and 81.2%, respectively, at 175 μg/cm^2^ after 48 h) were also different between EOs. It was also found that (*S*)-carvone, one of the identified compounds in AGEO, exhibited significant insecticidal activity.

The findings emphasize that carvone exhibits significant toxicity against a wide range of insect pests. Park et al. [128] indicated that (*R*)-carvone and (*S*)-carvone have considerable fumigant toxicity against the larvae of *L. ingenua*, in which 100% mortality was observed with a concentration of 0.005 mg/mL after 24 h. It was interesting that the same concentration of the synthetic insecticide Dichlorvos resulted in only 42% larval mortality. The insecticidal effects of carvone were also well-documented in the other related studies: probing and feeding deterrents to *M. persicae* [114], fumigant toxicity against *D. melanogaster* [164], toxicity, repellency, and acetylcholinesterase inhibitory to *S. oryzae* [112,131], contact and fumigant toxicity, repellency, antinutritional and acetylcholinesterase inhibitory against *S. zeamais* [85,115,165], toxicity and acetylcholinesterase inhibitory against *R. dabieshanensis* [120], fumigant toxicity against *C. chinensis* and *C. maculatus* [124], and repellency to *R. dominica* and *S. granarius* [166]. Furthermore, Abdelgaleil et al. [167] indicated that carvone can enhance the insecticidal potential of a natural pesticide, spinosad, against *S. oryzae*, in which 100% mortality of adults was attained in wheat treated with 0.5 mg/kg of spinosad + 2.0 g/kg of carvone after 21 days. Furthermore, no progeny of *S. oryzae* survived the combined treatment (0.5 mg/kg of spinosad + 2.0 g/kg of carvone) at 45 and 90 days.

Additionally, the acaricidal effects of carvone were also documented. For example, the acaricidal activity of (*S*)-carvone and (*R*)-carvone (with LC_50_ values of 527.1 and 285.4 ppm, respectively) against adult *T. urticae* was reported [148]. In the study of Sabahi et al. [168], the acaricidal effect of four terpenes, including carvone, citral, cineole, and limonene, against adult females of *V. destructor* was evaluated and compared with the synthetic acaricide tau-fluvalinate. The results indicated that carvone (4 h LC_50_ = 272.7 μg/mL), which did not differ significantly from tau-fluvalinate (4 h LC_50_ = 272.30 μg/mL), was more toxic to the mites than the other compounds. Furthermore, carvone had low toxicity to honeybee workers (*Apis mellifera* L.). They concluded that the high selectivity ratio of carvone for honeybees indicates its great potential for managing varroa mites.

Accordingly, the acaricidal and insecticidal potential of EOs may be due to the predominant presence of the monoterpene carvone. However, other dominant terpenes and/or low-quantity compounds can be toxic to the arthropod pests. Indeed, the biological activity of essential oils may be affected by their chemical profiles, ranging from the direct effects of the main compounds to the synergistic or antagonistic effects of minor ones.

### 4.3. Nematicidal Effects

Previous studies have demonstrated that the EOs exhibit promising nematicidal effects against harmful nematodes [169,170,171,172]. For example, the susceptibility of *the Panagrolaimus* genus, known for its ability to survive extreme drying, freezing, and cryptobiosis, to AGEO and MSEO was assessed by Zouh, with LC_50_ values of 0.4 and 0.5 µL/mL, respectively. Interestingly, the toxicity of AGEO and MSEO against *Panagrolaimus* sp. juveniles was better than that of several assessed EOs [173].

Based on the scope of the present review paper, the key findings regarding the nematicidal effects of the carvone-rich EOs of *C. carvi* and *M. spicata* are summarized in Table 4. The nematode groups most susceptible to these EOs belong to the order Tylenchida and the families Heteroderidae and Hoplolaimidae, which include some of the most destructive agricultural pests. For instance, most research on the nematicidal activity of these EOs has focused on root-knot nematodes of the genus *Meloidogyne*. Oka et al. [174] reported that the CCEO and MSEO, containing 50.0% and 58.0% carvone, respectively, exhibited significant nematicidal activity against *Meloidogyne javanica* (Treub) Chitwood (Tylenchida: Heteroderidae). The observed effects included increased immobility of second-stage juveniles (J2), reduced egg hatching, and fewer galls on cucumber plants. Treatment with 500 µL of either essential oil within 2 days resulted in 100% immobility of *M. javanica* J2, an efficacy that was significantly higher than that of the 21 other conventional EOs tested.

### 4.4. Herbicidal Activity

The herbicidal potential of carvone-rich EOs represents natural allelochemicals that negatively impact seed germination and seedling development by interfering with fundamental physiological processes [180]. The results of investigations focused on the herbicidal effects of the EOs are compiled in Table 5.

Extensive in vitro research has identified these compounds as potent pre-emergence tools, primarily through seed germination bioassays. Razavi et al. [99] demonstrated that (*R*)-carvone achieves the complete inhibition of *Amaranthus retroflexus* and *Portulaca oleracea* at concentrations exceeding 0.1 mg/mL, while Goudarzvande Chegini et al. [181] highlighted (*R*)-carvone as a highly potent inhibitor for *Echinochloa crus-galli* with an EC_50_ of 1.29 mM. These findings are supported by the model study of Vokou et al. [182], who, in a comprehensive study of 47 monoterpenoids on *Lactuca sativa*, ranked ketones—including both carvone stereoisomers and dihydrocarvone—as the most inhibitory class, resulting in seedling growth of less than 0.5% of the control. Among the EOs, caraway oil (CCEO) frequently exhibits the highest activity due to its significant (*S*)-carvone (63–71%) and limonene (25–35%) contents, effectively suppressing weeds such as *Sinapis arvensis* and *Sonchus oleraceus* [183]. Marichali et al. [184] also observed the complete inhibition of *Phalaris canariensis* at 100 µL/mL. Dill oil (AGEO) showed moderate to strong herbicidal effects. Hamidian et al. [185] found that it inhibited *A. retroflexus* germination by 67% at 600 µL/L, though it was less potent than EOs rich in carvacrol or thymol. It is particularly effective against parasitic weeds like *Cuscuta* spp., achieving 94–100% inhibition at 1% concentration [186]. Spearmint EO (MSEO), despite its lower carvone content, strongly inhibits *A. retroflexus* [183]. Rolli et al. [187] noted that it is more effective on seedlings than seeds, with significant root length inhibition (64.2%) at 1 mL/L.

The transition from laboratory assays to in situ and greenhouse evaluations has shifted the focus toward foliar application, post-emergence efficacy, and crop selectivity. Goudarzvande Chegini et al. [181] reported that (*R*)-carvone foliar sprays reduced shoot growth in barnyard grass by up to 83.3% at a 2% concentration. Synowiec et al. [188] observed that while CCEO showed short-term herbicidal effects against *Chenopodium album* and *Avena fatua*, its efficacy was lower than that of peppermint oil. Moreover, the short-term nature of these effects, due to rapid evaporation, remains a technical challenge [188]. A significant breakthrough in this field is the discovery of inherent crop selectivity. Synowiec et al. [189] and Rys et al. [190] demonstrated that caraway oil emulsions and bio-nanoemulsions can selectively target *E. crus-galli* in maize crops without compromising the biomass or efficiency of the photosynthetic apparatus of the *Zea mays* seedlings. This selectivity is mirrored in cereals, where Turgut and Coskun [191] observed high tolerance in wheat species to *Mentha piperita* EO, suggesting a niche for broadleaf weed control in grain production. Beyond field applications, (*S*)-carvone has also proven to be an effective sprout inhibitor for stored potatoes, performing as well as, or better than, synthetic IPC/CIPC mixtures when applied at regular 6-week intervals [192].

Carvone-rich EOs are established as potent botanical leads for both pre-emergence (germination inhibition) and post-emergence (foliar damage) applications. Carvone-rich oils exhibit multifaceted biological activity (mechanisms of action), as they disrupt mitochondrial respiration, inhibit DNA synthesis, compromise cell membrane integrity, and suppress photosystem II function, thereby preventing seedling growth [190,193] and making them valuable tools for resistance management. Despite the established herbicidal status of these oils, several technical problems persist, most notably the high volatility and environmental sensitivity of monoterpenes, which often lead to inconsistent field performance and necessitate high application doses [185]. Future perspectives center on adopting innovative delivery systems to overcome these limitations. The shift toward carvone–PLGA composites [90], emulsification [188], and nanoemulsification [191] represents a critical transition toward precision formulations. These advanced technologies enhance the stability of volatile components, improve leaf adherence, and increase the overall bioavailability. By transforming volatile EOs into stable, durable products, these formulations offer a viable path toward commercially competitive, environmentally safe bioherbicides for integrated crop protection.

**Table 5:** Herbicidal activity of carvone and carvone-rich essential oils against weeds and crops.

Weeds/Crops	EO: Main Compounds [%] [No. of EOs/Compounds Tested]	MIC or % Inhibition	Methods	Ref.
*Lactuca sativa*	(*R*)-carvone (*S*)-carvone [47 monoterpenes]	IR 88.8% at 2.5 µL 98.9 at 2.5 µL	In vitro seed germination bioassay on wetted filter paper in Petri dishes. 2.5 µL of EOs or control was applied to seeds.	[182]
*Alcea pallida* *Amaranthus retroflexus* *Centaurea salsotitialis* *Raphanus raphanistrum* *Rumex nepalensis* *Sinapis arvensis* *Sonchus oleraceus*	Carvone	IR 21.7% 76.2% 100% 100% 95.8% 98.7% 99.9%	In vitro seed germination bioassay on wetted filter paper in Petri dishes. Essential oil was tested at 4 concentrations of 3, 6, 10, and 20 µL. Distilled water was used as a control.	[183]
*Amaranthus retroflexus*, *Portulaca oleraceae*, *Medicago sativa* *Trifolium alexandrinum*	(*R*)-carvone and (*R*)-carvone-PLGA-composite	EC_100_ 0.1 mg/mL 0.1 mg/mL	In vitro seed germination bioassay at 4 concentrations: 0.001, 0.01, 0.1, and 1 mg/mL. Distilled water was used as a control.	[98]
*Solanum tuberosum*	(*S*)-carvone	Carvone was able to suppress sprout growth during the whole storage period	Application of 50 to 100 mL of carvone per 1000 kg of potatoes at 1/6/9-week intervals during storage. Carvone treatment was compared with IPC/CIPC, a commercial sprout inhibitor.	[192]
*Echinochloa crus-galli*	(*R*)-carvone [7 monoterpenes]	Seed germination EC_50_ 1.29 mM IR 36.8% at 1mM, 73.7% at 2mM Shoot growth EC_50_ 0.96 mM Root growth EC_50_ 0.3 mM Shoot growth (two-leaf stage)-IR 81.0%, 83.3%	In vitro seed germination assay at 6 concentrations of 1, 2, 3, 4, 6, and 8 mM. In situ foliar application with 2 concentrations of 1 and 2% of essential oil emulsions on weeds at the two-leaf stage. Distilled water containing DMSO (0.5% *v*/*v*) and Triton X-100 (0.02%) was the control.	[194]
*Raphanus sativus* *Lepidium sativum*	(*R*)-carvone [27 monoterpenes]	Seed germination IR 96% at 10^−3^ M Radicle elongation IR 79% at 10^−3^ M	Two methods: (1) dipping assessed antigerminative activity and (2) volatilization using 4 different concentrations of 10^−6^, 10^−5^, 10^−4^, 10^−3^ M. The control and negative control treatments were not indicated.	[195]
*Alcea pallida* *Amaranthus retroflexus* *Centaurea salsotitialis* *Raphanus raphanistrum* *Rumex nepalensis* *Sinapis arvensis* *Sonchus oleraceus*	CCEO: (*S*)-carvone, limonene Composition not reported	IR 53.3% 92.6% 92.4% 100.0% 86.4% 100.0% 100.0%	In vitro seed germination bioassay on wetted filter paper in Petri dishes. Essential oil was tested at 4 concentrations: 3, 6, 10, and 20 µL. Distilled water was used as a control.	[189]
*Chenopodium album* *Avena fatua* (ES)	CCEO: carvone 63.2, limonene 34.8 [2 EOs]	It showed short-term herbicidal effects against the weeds, but PPEO was generally more herbicidal than CCEO	Foliar application of 4.25 g of essential oil emulsions at the 4–5 leaf stage for *C. album* and 2–3 leaf stage for *A. fatua.* The control plants were treated with water only.	[183]
*Echinochloa crus-galli* *Chenopodium album*	CCEO: carvone 63.2, limonene 34.8 [3 EOs encapsulated in maltodextrin]	MIC 200 kg/ha IR 63.5% at 200 kg/ha	EOs at 3 concentrations (0.75, 1.5, and 3 g per pot) were spread on the pot surface and mixed into the soil substrate. Two control treatments were used: soil substrate only and the soil substrate with MDX.	[196]
*Avena fatua* *Bromus secalinus* *Amaranthus retroflexus* *Centaurea cyanus*	CCEO: carvone 63.2, limonene 34.8 [12 EOs]	ED_50_ 0.2 g/L 0.17 g/L 0.04 g/L 0.04 g/L	In vitro seed germination bioassay on wetted filter paper in Petri dishes at 5 concentrations of each oil, 0.2, 0.6, 1.2, 2.4, and 7.2 g/L. The control treatment contained only water and acetone.	[197]
*Echinochloa crus-galli*	CCEO: carvone, 66.4, limonene 32.5 [2 EOs]	CCEO was effective on barnyard grass, causing leaf injuries and a reduction in biomass	Foliar application of 2 EO concentrations of 2.5 and 5% at the 3–4 leaves stage. The control plants were hand-sprayed with water only and water + adjuvant.	[189]
*Echinochloa crus-galli*	CCEO: carvone 63.3, limonene 35.2	ED_10_ at 2.0% ED_50_ at 5.0% ED_90_ at 13.5%	Foliar application of 5 concentrations, i.e., 1, 1.5, 2, 5, and 10%, of bio-nanoemulsions at the three-leaf stage. Three control treatments: surfactant only, distilled water only, and a commercial mixture of herbicides.	[190]
*Phalaris canariensis*	CCEO: carvone 71.1, limonene 25.4	IR 100% at 100 µL/mL	In vitro seed germination bioassay on wetted filter paper in Petri dishes with EOs at 5 concentrations of 5, 10, 50, 75, and 100 µL/mL. Control only the treatment of the water–methanol mixture.	[184]
*Amaranthus retroflexus*	AGEO: carvone 59.2, limonene 14.2, cis-dihydrocarvone 6.3, trans-dihydrocarvone 7.5 [7 EOs]	IR 70% at 100 µL/L 80% at 300 µL/L 67% at 600 µL/L	Germination inhibition test at 3 concentrations of 100, 300, and 600 µL/L of nanoemulsions of the EOs. Foliar application of 2000 µL/L of EOs at the 4–6 leaf stage. Control was distilled water only and distilled water + Tween 80.	[185]
*Solidago canadensis*	AGEO: carvone 58.4, limonene 35.8 [6 EOs]	ED 0.25 mg/mL	In vitro seed germination bioassay on wetted filter paper in Petri dishes with 6 concentrations of 2.5, 1.25, 0.625, 0.25, 0.125, 0.062 mg/mL. Negative controls were distilled water and acetone.	[193]
*Cuscuta campestris* *Cuscuta epithymum* *Medicago sativa* *Trifolium pratense*	AGEO: carvone 51.7, limonene 39.9	IR 100% at 1% 94% 100% 100%	In vitro seed germination bioassay on wetted filter paper in Petri dishes with 4 concentrations of 1, 0.5, 0.1, and 0.01% of essential oil. Water + Tween 20 was used as a control.	[192]
*Sorghum halepense*	AGEO: carvone 40.5, limonene 32.2	Seed germination—IR 61.5% at 500 µL Shoot length 38.2% at 5 mL	In vitro seed germination bioassay on wetted filter paper in Petri dishes, measuring seed germination and shoot length using 5 mL of essential solution. The control solution was not indicated.	[198]
*Alce apallida* *Amaranthus retroflexus* *Centaurea salsotitialis* *Sinapis arvensis* *Sonchus oleraceus* *Raphanus raphanistrum* *Rumex nepalensis*	MSEO: (−)−carvone, limonene	IR 53.3% 97.1% 89.4% 99.0% 94.4% 100.0% 93.3%	In vitro seed germination bioassay on wetted filter paper in Petri dishes. Essential oil was tested at 4 concentrations: 3, 6, 10, and 20 µL. Distilled water was used as a control.	[184]
*Solanum lycopersicum*	MSEO: carvone 47.4, β-phellandrene 11.3, menthol 5.5	IR 10.3% at 1 mL/L (germination) 64.2% (root length) 48.4% (shoot length)	In vitro seed germination bioassay on wetted filter paper in Petri dishes. Two control groups: (1) distilled water alone and (2) a solution of Tween 20. Essential oil was suspended in distilled water at 1000 mg/L. Seedling growth experiments were carried out on ¼-strength MS medium, solidified with 0.8% (*w*/*v*) agar, and 10 μL of essential oil emulsion was used.	[188]
*Triticum durum* *Triticum spelta* *Triticum aestivum* *Triticum monococcum* *Triticum dicoccum*	MSEO: carvone 62.9, limonene 8.2, cis-dihydrocarvone 5.6, 1,8-cineole 5.4 [2 EOs]	Mean germination reduction—14.97%	In vitro seed germination bioassay on wetted filter paper in Petri dishes at 3 concentrations of 0.2, 0.4, and 0.8 μL of essential oil. Water was used as a control.	[192]

MIC—minimum inhibitory concentration, IR—inhibition rate, EC—effective concentration, ED—effective dose, CCEO—*Carum carvi* essential oil, MSEO—*Mentha spicata* essential oil, AGEO—*Anethum graveolens* essential oil.

## 5. Summary

Based on the available research, carvone and carvone-rich essential oils (EOs) show high efficacy against diverse fungal and bacterial plant pathogens, phytophagous invertebrates, and weeds. These natural compounds offer significant potential for managing diseases before and after harvest. As a result, they represent a viable alternative to synthetic chemicals. One clear advantage is that these EOs are generally recognized as safe (GRAS) and carry a low risk for microbial resistance development. This makes them ideal candidates for integrated pest management systems.

In diverse biological assays, the stereochemistry of carvone strongly affects its efficacy. The (R)-enantiomer (L-carvone or (-)-carvone) usually shows stronger biological activity than the (S)-enantiomer. For antifungal activity, (R)-carvone often achieves the complete inhibition of pathogens, such as *Geotrichum citri-aurantii*. In contrast, the (S)-isomer usually shows much lower activity. As a result, essential oils containing (R)-carvone (such as MSEO and other mint EOs) are more effective than those with the (S)-enantiomer (such as CCEO and AGEO). This strength also appears in acaricidal and insecticidal tests. For example, (R)-carvone is more toxic to the two-spotted spider mite (*Tetranychus urticae*) and acts as a highly effective synergist. Its herbicidal activity is also strong, especially against weed germination and root growth, such as barnyard grass (*Echinochloa crus-galli*). However, this activity can be species-specific. For example, (S)-carvone is more effective in some tests, such as *Lactuca sativa* seed bioassays, and both isomers can have identical minimum inhibitory concentrations (MICs) for certain microorganisms.

Several challenges restrict the commercial use of carvone-rich EOs. The main issue is the lack of comparability between research results. This lack of standardization results from a complex mix of technical, biological, and chemical factors. Methodological differences, such as comparing inhibition zones in diffusion methods with MIC values from macro- or microdilution methods, yield conflicting results. The results are also affected by the biological variability of target organisms, their life stages, and the unique chemical makeup of EOs from different plant parts or chemotypes. Technical challenges, such as differences in microorganism sensitivity to lab solubilizers (e.g., ethanol versus Tween) and variations in bioassay protocols, further complicate data comparison. For example, larval fumigant toxicity tests versus adult contact toxicity tests use different units for EO concentrations, making the synthesis of global data difficult.

While searching the literature, we found many articles relevant to this topic that were unsuitable for citation. Many were of low quality or contained serious errors. The first group of articles did not provide the composition of the EO used. Such articles should not be published. The second group had mistakes in identifying the plant from which the oil was isolated. Worse, some of these mistakes appeared in several review articles. These problems result from the absence or poor use of research standards. Every study on the biological activity of EOs should include a thorough analysis of their composition.

In addition to research barriers, practical use faces hurdles. High volatility, low polarity, and sensitivity to oxygen, light, and humidity all limit their application.

Recent advancements in formulation science offer promising solutions to these limitations by improving the stability, bioavailability, and efficacy of EOs. Currently, nanoemulsification and nanoencapsulation are the most effective techniques for agricultural applications [199]. Encapsulation in chitosan is now the most frequently used method [200]. These approaches have proven successful in recent studies on pure carvone and carvone-rich EOs, such as caraway, dill, and spearmint oils. New delivery systems enhance the stability and effectiveness of volatile components, protect against evaporation and environmental harm, and improve bioavailability. They also enable controlled release, so lower doses are required than with pure EOs. Importantly, encapsulated EOs used for fruit and vegetable preservation do not alter the organoleptic properties of the produce.

In summary, carvone-rich EOs are a promising and safe alternative to synthetic pesticides. They have a good environmental record and carry less risk of resistance. Still, challenges remain. Most studies are lab-scale in vitro or in situ experiments with pure EOs. Although the results are promising, there are not enough studies on how new EO formulations perform in real-world settings. In addition to developing standard tests, more field trials are needed. This is vital for cost-effective, energy-efficient, biodegradable EO formulations.

## Figures and Tables

**Table 4:** Nematicidal effects of carvone-rich essential oils.

Nematode(s) and Stage	EO: Main Components [%]	Nematicidal Effects	Methods	Ref.
*Meloidogyne javanica*, J2s and eggs	CCEO: carvone 50.0, limonene 48.0	J2 immobility and reduction in egg hatching at 800 and 600 μL/L after 2 and 7 days, respectively A gall reduction on cucumber at 200 mg/kg of soil	Eggs and J2s’ water were exposed to EO solutions and incubated at 25 °C. EO diluted in ethanol was mixed with nematode-infested sandy soil in plastic pots	[174]
*R. reniformis*, juveniles and adults *Criconemella* spp., juveniles and adults *Hoplolaimus* spp., juveniles and adults *Meloidogyne incognita*, eggs	MSEO: carvone 58.1, phyllandrene 6.0, limonene 6.0, pulegone 4.4, menthone 4.0	100% immortality after 72 h and 92.2% egg hatching reduction at a concentration of 0.1%	All soil stages were separately transferred to the EO solutions in Petri dishes	[175]
*Meloidogyne javanica*, J2s and eggs	MSEO: carvone 58.0, limonene 19.0	J2 immobility and reduction in egg hatching at 800 and 600 μL/L after 2 and 7 days, respectively	Eggs and juveniles’ water were exposed to EO solutions and incubated at 25 °C	[174]
*Meloidogyne incognita*, juveniles	MSEO: carvone 60.4, dihydrocarvone 8.4, neodihydrocarveol 3.3, limonene 3.0	Lethality with 72 h-EC_50_ = 358.0% mg/L	Juveniles were treated with separate solutions of EO in plate wells	[176]
*Meloidogyne javanica*, J2 and eggs	MSEO: carvone 71.9, limonene 14.3, caren-4-ol isomer 3.2	Lethality of J2s (72 h-LC_50_ = 0.2 mg/mL) and egg hatching inhibitory (60.6% at a concentration of 1 μg/μL after 28 days)	Eggs and juveniles were treated with separate solutions of EO in plate wells	[177]
*Meloidogyne hapla*, eggs	MSEO: carvone 27.1, menthol 26.4, isomenthone 14.4, 1,8-cineole 7.1, limonene 5.3	23.8% egg hatching inhibition at 4 μL/mL after 7 days	Eggs were treated with separate solutions of EO in Petri dishes	[178]
*Meloidogyne javanica*, J2s	MSEO: carvone 66.3, *ρ*-cymene 7.4, limonene 5.0, dihydrocarveol 3.8	100% mortality by the prepared nanoemulsion of EO at 4000 ppm after 48 h	Juvenile suspension was placed into screw-capped tubes containing various rates of EO and incubated at 26 °C for 3 days	[179]

Scientific and common names of nematodes: *Meloidogyne hapla* Chitwood (Tylenchida: Heteroderidae) (Northern root-knot nematode); *Meloidogyne incognita* (Kofoid and White) Chitwood (Tylenchida: Heteroderidae) (Southern root-knot nematode); *Meloidogyne javanica* (Tylenchida: Heteroderidae) ((Treub) Chitwood) (Javanese root-knot nematode); *Rotylenchulus reniformis* Linford and Oliveira (Tylenchida: Hoplolaimidae) (the reniform nematode). EC_50_ indicates 50% effective concentration (death/inhibitory/immotility).

## Data Availability

No new data were created or analyzed in this study. Data sharing is not applicable to this article.
